# Intramolecular Telomeric G-Quadruplexes Dramatically Inhibit DNA Synthesis by Replicative and Translesion Polymerases, Revealing their Potential to Lead to Genetic Change

**DOI:** 10.1371/journal.pone.0080664

**Published:** 2014-01-14

**Authors:** Deanna N. Edwards, Amrita Machwe, Zhigang Wang, David K. Orren

**Affiliations:** 1 The Graduate Center for Toxicology, University of Kentucky College of Medicine, Lexington, Kentucky, United States of America; 2 Markey Cancer Center, University of Kentucky College of Medicine, Lexington, Kentucky, United States of America; Université de Sherbrooke, Medicine, Canada

## Abstract

Recent research indicates that hundreds of thousands of G-rich sequences within the human genome have the potential to form secondary structures known as G-quadruplexes. Telomeric regions, consisting of long arrays of TTAGGG/AATCCC repeats, are among the most likely areas in which these structures might form. Since G-quadruplexes assemble from certain G-rich single-stranded sequences, they might arise when duplex DNA is unwound such as during replication. Coincidentally, these bulky structures when present in the DNA template might also hinder the action of DNA polymerases. In this study, single-stranded telomeric templates with the potential to form G-quadruplexes were examined for their effects on a variety of replicative and translesion DNA polymerases from humans and lower organisms. Our results demonstrate that single-stranded templates containing four telomeric GGG runs fold into intramolecular G-quadruplex structures. These intramolecular G quadruplexes are somewhat dynamic in nature and stabilized by increasing KCl concentrations and decreasing temperatures. Furthermore, the presence of these intramolecular G-quadruplexes in the template dramatically inhibits DNA synthesis by various DNA polymerases, including the human polymerase δ employed during lagging strand replication of G-rich telomeric strands and several human translesion DNA polymerases potentially recruited to sites of replication blockage. Notably, misincorporation of nucleotides is observed when certain translesion polymerases are employed on substrates containing intramolecular G-quadruplexes, as is extension of the resulting mismatched base pairs upon dynamic unfolding of this secondary structure. These findings reveal the potential for blockage of DNA replication and genetic changes related to sequences capable of forming intramolecular G-quadruplexes.

## Introduction

**Figure 1:**
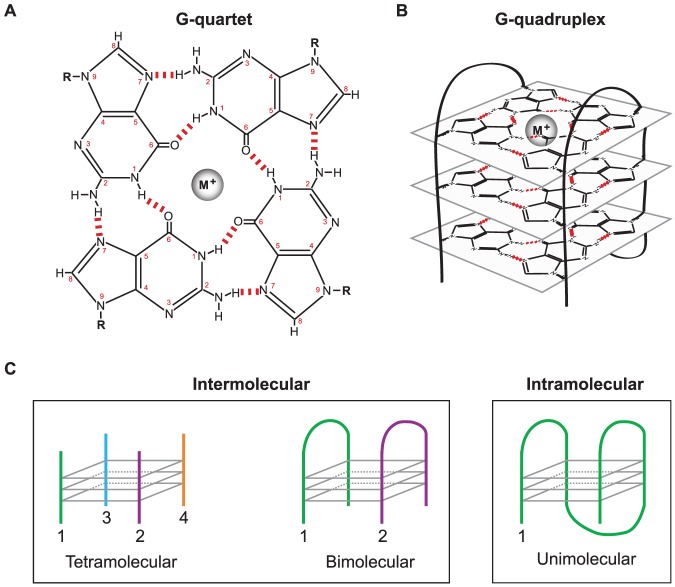
G-quadruplex Structure. A) Structure of a G-quartet in the presence of a monovalent cation (M^+^). Hydrogen-Hoogsteen bonds are indicated by red dashes. B) An intramolecular G-quadruplex structure, consisting of three planar G-quartets. C) G-quadruplex classifications, based on the number of strands involved.

G-rich sequences including those present in telomeres present numerous difficulties for the replication machinery. Each human telomere is composed of 5–15 kbp of the duplex hexameric repeat sequence TTAGGG/AATCCC, ending in single-stranded 3′ overhangs of the G-rich strand. Notably, telomeric TTAGGG repeats and many other G-rich regions possess the potential to form G-quadruplexes, compact secondary structures that can assemble through interactions between four runs of at least two to three guanine nucleotides. Specifically, Hoogsteen interactions between the N^2^, N^7^, N^1^ and O^6^ positions among four guanine bases form a G-quartet, a structure stabilized by a monovalent cation such as K^+^ or Na^+^ (Fig. 1A), and stacking of two or more G-quartets forms a G-quadruplex; a G-quadruplex comprised of three G-quartets is depicted in Fig. 1B. Furthermore, G-quadruplexes can be defined by the number of strands involved; intermolecular G-quadruplexes form from the interactions of two or more DNA strands while intramolecular G-quadruplexes assemble by folding of a single DNA strand (Fig. 1C). Depending upon the nature of the guanine runs and surrounding sequences involved, both intra- and intermolecular G-quadruplexes can be relatively stable secondary structures [1].

Although G-quadruplexes are readily formed *in vitro* by a variety of G-rich sequences, including the Fragile X syndrome-associated CGG repeats [2], [3], the c-myc promoter sequence [4], and the human telomeric sequence [5], [6], [7], detection of these structures *in vivo* has been challenging. However, evidence indicates that G-quadruplexes form in *Saccharomyces cerevisiae* [8] and the macronuclear telomeres of the ciliated protozoa *Stylonychia lemnae* [9]. Very recently, Balasubramanian and colleagues have successfully visualized formation of G-quadruplex-specific foci at telomeric and non-telomeric locations in fixed human cells and shown that the number of G-quadruplex foci increased during DNA replication [10]. Based on sequence analyses, more than 376,000 sites within the human genome exhibit the potential to form G-quadruplexes; most of these are concentrated in telomeric regions and gene promoters, with G-quaduplex formation in promoters being thought to influence transcriptional regulation [11], [12], [13]. G-quadruplex formation might be promoted under circumstances in which single-stranded regions are revealed such as during transcription, replication, or recombination or within telomeric overhangs [1]. Among these single-stranded regions, intramolecular G-quadruplexes may be more likely to form *in vivo* than intermolecular G-quadruplexes [1], [14].

Stable secondary structures including intramolecular G-quadruplexes may impede replication. As a strong indicator of replication difficulties at G-rich telomeric sequences, human telomeres develop double-strand breaks dependent upon replication and thus resemble fragile sites [15]. Specifically within telomeres, replication fork stalling caused by G-quadruplexes may lead to telomere instability that can either increase overall genome instability and cancer susceptibility or lead to checkpoint-mediated apoptosis or senescence that might contribute to other features of aging. In fact, more double-strand breaks occurred following treatment with a G-quadruplex-stabilizing ligand in cells lacking the translesion polymerases pol η or pol κ, not only indirectly suggesting the existence of G-quadruplexes *in vivo* but also implying that these structures both inhibit replicative polymerases and are processed subsequently by translesion polymerases [16]. *In vitro* biochemical experiments also showed a bimolecular G-quadruplex on the template strand inhibits pol δ, the main polymerase in lagging strand synthesis [17]. However, such multi-stranded G-quadruplexes differ substantially in structure from one another and from intramolecular G-quadruplexes that are probably formed *in vivo*. In addition, direct examination of the effects of G-quadruplexes on DNA synthesis by translesion polymerases are lacking. Therefore, we set out to test the effect a defined intramolecular G-quadruplex (formed from human telomeric sequence) on the template strand would have on a range of human and non-human DNA polymerases. In these experiments, we tested not only replicative polymerases but also translesion DNA polymerases that are widely thought to function in response to stalled replication. With all DNA polymerases tested here, our results indicate that intramolecular G-quadruplexes are significant obstacles to DNA synthesis and have the potential to cause not only replication blockage and collapse but also misincorporation and mutagenesis.

## Materials and Methods

### Enzymes

T4 polynucleotide kinase, Klenow fragment, 3′ to 5′ exonuclease-deficient (hereafter abbreviated as Kexo^−^) and T4 DNA polymerase were purchased from New England Biolabs (Ipswich, MA); exonuclease-deficient T4 DNA polymerase was from Lucigen (Middleton, WI). Human polymerase δ, a gift from Guo-Min Li (University of Kentucky), was purified as described [18]. All other purified DNA polymerases used, including human polymerase η, *S. cerevisiae* polymerase η, human polymerase κ, human polymerase β, and human polymerase μ, were acquired from Enzymax (Lexington, KY).

### DNA Substrates

**Figure 2:**
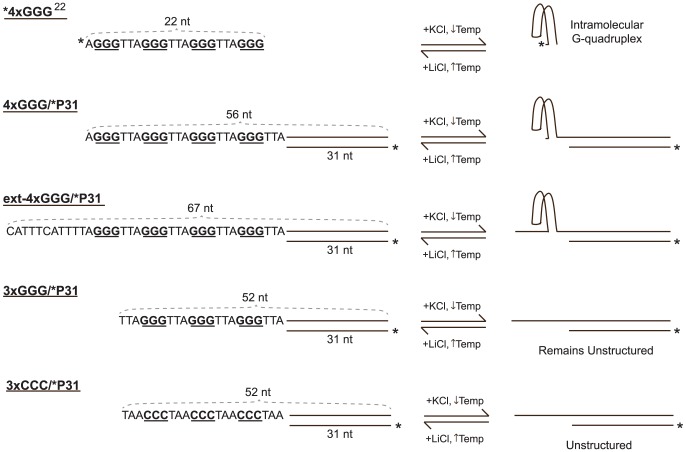
Primer/Template Substrates. To generate primer/template substrates, a template strand (4×GGG, ext-4×GGG, or 3×GGG) is annealed to a 5′ radiolabeled primer strand, *P31 or *P34 (not shown). The 4×GGG^22^ oligomer (top), which is capable of forming an intramolecular G-quadruplex, was used as a model for the single-stranded template regions of 4×GGG/*P31 and ext-4×GGG/*P31. Conditions favoring (or disfavoring) G-quadruplex formation are indicated; notably, these conditions do not affect the structure of the template regions of control 3×GGG/*P31 and 3×CCC/*P31 substrates (also see Fig. 3C, Fig. 4, and Fig. S1).

DNA oligonucleotides purified by polyacrylamide gel electrophoresis (PAGE) were obtained from Integrated DNA Technologies (Coralville, IA). Sequences of all oligos are shown in Table S1. Importantly, the template oligos 4×GGG and ext-4×GGG contain four human telomeric repeats, and thus four runs of three consecutive guanines that could potentially fold into an intramolecular G-quadruplex. However, the oligo 3×GGG contains only three human telomeric repeats and is not capable of forming an intramolecular G-quadruplex. The oligo 4×GGG^22^ reflecting only the telomeric template region of 4×GGG and ext-4×GGG was a gift from Michael Fried (University of Kentucky). To generate the substrates used, 4×GGG^22^ and primer oligos P31 and P34 were 5′ radiolabeled using ^32^P-γ-ATP and T4 polynucleotide kinase. Radiolabeled oligomers (hereafter indicated by asterisks) were passed through Mini Quick Spin Oligo Columns (Roche, Indianapolis, IN) to remove unincorporated ATP. Labeled primer/template substrates (Fig. 2) were produced by annealing 3×CCC, 3×GGG, 4×GGG, or ext-4×GGG to *P31 or *P34 in 50 mM Tris-HCl (pH 8.0) and 10 mM MgCl_2_ after heating to 90°C and slow cooling to room temperature. Substrates were then purified by native PAGE (10%), excised, and eluted into 10 mM Tris-HCl (pH 8.0) containing 10 or 25 mM NaCl.

### Dimethyl Sulfate Protection Assays

In 0 or 75 mM KCl, *4×GGG^22^ and 3×GGG oligomers (1.75 and 0.3 nM, respectively) were heat-denatured at 90°C and slow cooled to room temperature, with added KCl theoretically promoting G-quadruplex formation. Samples were treated with 0.05% dimethyl sulfate (DMS) (Sigma-Aldrich, St. Louis, MO) in extension buffer [40 mM Tris-HCl (pH 8.0), 1 mM MgCl_2_, 5 mM dithiothreitol, 100 µg/ml bovine serum albumin (BSA), 0.1% NP40, and 250 µM ATP] in 0 or 75 mM KCl at 25°C for 10 min, and the reaction was stopped by addition of 750 mM β-mercaptoethanol and 1.125 M sodium acetate (pH 7.0). DNA from each sample was collected by standard ethanol precipitation using yeast tRNA (10 µg) as carrier. The resulting pellet was resuspended in 10% piperidine (Sigma-Aldrich), incubated at 90°C for 30 min, and the liquid was removed using vacuum evaporation. The samples were resuspended in 10 mM Tris (pH 8.0) and an equal volume of formamide loading buffer (95% formamide, 20 mM EDTA, 0.05% bromophenol blue, and 0.05% xylene cyanol) was added. To facilitate precise comparisons between samples, equal amounts of radioactivity in individual samples were analyzed by denaturing PAGE (14%). Labeled DNA fragments were visualized using Storm 860 Phosphorimager and ImageQuant software (GE Healthcare).

### DNA Structure Assessment by Native PAGE

3×CCC/*P31, 4×GGG/*P31, and 3×GGG/*P31 (200 pM each) were incubated in 75 mM KCl or LiCl at 25°C for 1 h to allow for secondary structure formation, and subsequently one-sixth volume of acrylamide running dyes (30% glycerol, 0.25% xylene cyanol, 0.25% bromophenol blue) was added. Promotion of intermolecular G-quadruplex formation was accomplished by incubating 3×GGG/*P31 or 4×GGG/*P31 substrate (0.3 nM) with 3×GGG or 4×GGG olignonucleotide (6.7 µM), respectively, in 10 mM Tris (pH 8,0), 1 mM EDTA, and 1 M NaCl at 37°C for 42 h. Unless otherwise noted, DNA structures were electrophoresed by native PAGE (15%, 37.5∶1) at room temperature in 1× TBE (90 mM Tris-borate, 2 mM EDTA) containing either 75 mM KCl or LiCl in both the gel and running buffer. Labeled DNA products were visualized as described above.

### Primer Extension Assays

Primer/template substrates (0.2–0.4 nM) were treated with a DNA polymerase (Kexo^−^, exonuclease-proficient or -deficient T4 DNA polymerase, human polymerase δ, human polymerase η, *S. cerevisiae* polymerase η, human polymerase κ, human polymerase μ, or human polymerase β) at the specified concentrations in extension buffer with 75 mM KCl or LiCl and dNTPs (100 µM each) as indicated. Polymerase concentrations to be used were determined by the amounts needed to achieve full extension (or, in the case of pol μ and pol β, acceptable extension through the first GGG run) on control substrates not capable of forming intramolecular G-quadruplexes. Reactions were incubated for 5–60 min at 18–37°C with one or more dNTP's and/or in a stepwise manner as specified in the Results, and stopped using an equal volume of formamide loading buffer. Samples were heat-denatured and analyzed by denaturing PAGE (14%). Labeled primer strand DNA species were visualized as indicated above. For substrates containing the *P31 primer, quantitation regarding incorporation of nucleotides by individual polymerases and inhibitory effects of intramolecular G-quadruplexes was accomplished by comparing the amounts of individual or combined DNA species to the total amount of extended products (excluding unextended primers) for each lane. Product levels for substrates containing the *P34 primer were calculated by comparing individual or combined DNA species to total radioactivity for that lane, with minimum detection limits for DNA species set at three times the standard deviation of the mean of the background for each image. Where indicated, statistical significance was determined by Student's T-tests, two-tailed distribution paired analysis.

## Results

### Formation and stability of telomeric intramolecular G-quadruplex structures

**Figure 3:**
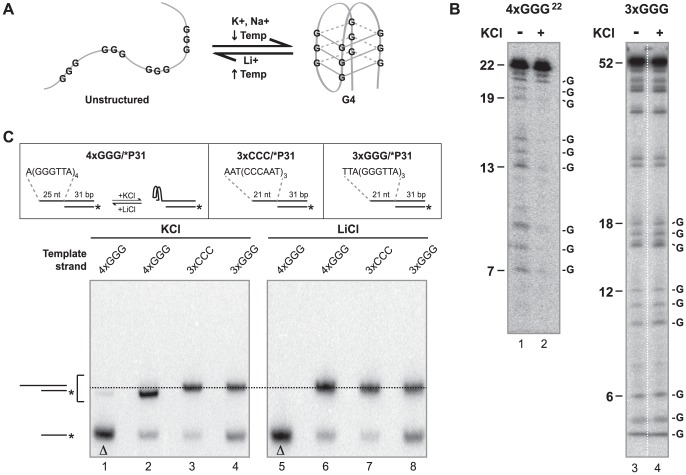
Template Sequence of 4×GGG/*P31 Forms an Intramolecular G-quadruplex. A) Intramolecular G-quadruplex formation from single-stranded DNA containing four GGG runs. Conditions that impact formation and dissociation of G-quadruplexes are indicated. B) To detect G-quadruplex formation, a DMS protection assay was performed on *4×GGG^22^ (lanes 1 and 2) or 3×GGG (lanes 3 and 4) in extension buffer conditions plus or minus 75 mM KCl as indicated. Products resulting from cleavage at unmethylated guanine bases are analyzed by denaturing PAGE. Representative lengths and positions of key guanines (G) for DNA fragments are indicated. C) Primer/template substrates (4×GGG/*P31, 3×CCC/*P31 and 3×GGG/*P31, pictured at top) were incubated in 75 mM KCl or LiCl for 1 h at 25°C. The structures of these substrates were analyzed by their relative migration on native PAGE (15%) containing 75 mM KCl or LiCl in the gel matrix and running buffer. For lanes 1 and 5, 4×GGG/*P31 substrate was heat-denatured before electrophoresis. The dotted line indicates migration of primer/template substrates with unstructured templates.

Intramolecular G-quadruplexes are formed by self-association of single DNA molecules through Hoogsteen interactions of four runs of two or more guanines in the presence of K^+^ or Na^+^ (Figs. 1 and 3A). These compact secondary structures are capable of forming from telomeric repeats or other G-rich sequences and may present problems for DNA polymerases during replication. In this study, we constructed primer-template DNA substrates with template strands containing telomeric G-rich repeats capable of assembling into G-quadruplex structures and subsequently examined DNA synthesis on these substrates by a variety of replicative and translesion polymerases. It was first necessary to determine whether and under what conditions the template strand of our primer-template substrates formed G-quadruplexes. We used several methods to detect G-quadruplex formation, including the DMS protection assay, DNA structure analysis using native PAGE, and primer extension assays under conditions that favor or disfavor G-quadruplex formation. The DMS protection assay is based on the Maxam-Gilbert sequencing protocol. DMS methylates the N^7^ position of guanine bases, but not guanines involved in a G-quadruplex structure (see Fig. 1A) [19], [20]. The DNA backbone is randomly cleaved by piperidine at methylated guanines generating a ladder-like pattern of DNA fragments when analyzed by denaturing PAGE [21]. Guanines involved in a G-quadruplex structure remain unmethylated and thus no backbone cleavage occurs at those positions [22]. The radiolabeled oligonucleotide *4×GGG^22^ is comprised of 22 nt of G-rich telomeric sequence containing four runs of three guanine bases and thus is capable of forming a G-quadruplex in the presence of K^+^ or Na^+^. Therefore, the DMS assay was performed on *4×GGG^22^ at 25°C in extension buffer without or with 75 mM KCl, and the resulting patterns of DNA fragments were compared after denaturing PAGE. In the absence of KCl, bands corresponding to DNA fragments cleaved at guanine bases were generated, indicating all guanine bases were methylated and G-quadruplex structures were not formed under these conditions (Fig. 3B, lane 1). Conversely, the intensities of these bands are dramatically reduced in the presence of 75 mM KCl (Fig. 3B, lane 2), clearly indicating a loss of DMS methylation at these guanines. This result demonstrates that telomeric sequence containing four G runs forms a G-quadruplex structure in the presence of 75 mM KCl, lower than the purported physiological K^+^ concentration [23]. In contrast, DMS assays on the 3×GGG 52-mer with telomeric sequence at its 5′ end showed identical patterns in 0 and 75 mM KCl, including prominent bands corresponding to its three GGG runs (Fig. 3B, lanes 3–4), indicating little or no G-quadruplex structure formation in either 0 or 75 mM KCl. While both substrates have the potential to form intermolecular G-quadruplexes, only the 4×GGG^22^ oligomer can assemble into an intramolecular structure. Thus, even though this assay cannot differentiate between inter- and intramolecular G-quadruplexes, these results suggest that the structure assumed by the 4×GGG^22^ substrate is likely to be an intramolecular G-quadruplex. Importantly, the telomeric sequences contained in these oligomers are identical to those within single-stranded template regions of primer-template substrates constructed with 4×GGG, ext-4×GGG, and 3×GGG oligomers (see Fig. 2) used below for structure analysis as well as for primer extension assays.

DNA structure analysis using native PAGE allows differentiation of intra- and intermolecular G-quadruplexes from one another and from unfolded structures. Intramolecular G-quadruplexes assume more compact structures that migrate faster than single-stranded DNA [22], [24]. Intermolecular G-quadruplexes also form a compact structure, but are composed of two or more strands and thus migrate much slower compared to single-stranded DNA [20], [25]. Therefore, we analyzed the electrophoretic migration of several primer-template substrates using differing monovalent cations in both the sample and electrophoresis buffers. We used buffers containing KCl, which promotes and stabilizes G-quadruplex formation, or LiCl which favors unstructured DNA (Fig. 3A) [22]. Although the duplex regions of these primer-template substrates were identical in length (31 bp) and sequence, the single-stranded template region of the test substrate 4×GGG/*P31 (Figs. 2 and 3C) is 25 nt and contains four runs of guanines (as for *4×GGG^22^) and maintains the potential to form intra- (Fig. 3A) or intermolecular G-quadruplexes in buffers containing K^+^ or Na^+^. Conversely, a control substrate 3×CCC/*P31, contains three telomeric C-strand repeats and thus entirely lacks guanines within the single-stranded (21 nt) template, ruling out the formation of any type of G-quadruplex (Figs. 2 and 3C); the single-stranded template region also lacks self-complementarity, excluding development of other Watson-Crick secondary structures. Another comparative control, the 3×GGG/*P31 substrate, contains three guanine runs in the single-stranded (21 nt) template (Fig. 2 and 3C); this design eliminates the possibility of an intramolecular G-quadruplex, although multiple template strands could potentially associate to form intermolecular G-quadruplexes. When these primer-template substrates were incubated and analyzed by native PAGE in buffers containing either 75 mM KCl or LiCl (Fig. 3C), the relative migration positions of both partial duplex control substrates, 3×CCC/*P31 and 3×GGG/*P31, were essentially identical to one another and unchanged in KCl versus LiCl (Fig. 3C, compare lanes 3, 4, 7, and 8 and position of dashed line). The substrate 4×GGG/*P31 migrated to a similar position as the control substrates in the presence of LiCl (Fig. 3C, lanes 6–8), suggesting the template exists in single-stranded form under these conditions. However, in KCl, the 4×GGG/*P31 substrate migrates at a somewhat faster rate than the control substrates (Fig. 3C, compare lanes 2–4), indicating that its template region, which is actually 4 nt longer than either control template, forms a more compact structure. The faster migration of the 4×GGG/*P31 substrate specifically in 75 mM KCl strongly suggests that the template strands fold into intramolecular G-quadruplexes under those conditions; slower migrating complexes were not detected in these gels (Fig. 3C). In contrast, samples containing much higher 3×GGG or 4×GGG oligomer concentrations combined with 3×GGG/*P31 or 4×GGG/*P31 substrate, respectively, incubated under high Na+ ion conditions did reveal slower migrating bands consistent with formation of intermolecular G-quadruplexes containing both labeled substrate and at least one unlabeled oligomer (Fig. S1). Notably, amounts of the slower migrating bands were much greater using 3×GGG/*P31 substrate plus 3×GGG than with 4×GGG/*P31 plus 4×GGG (Fig. S1, compare lanes 2 and 3). This is probably because most of the 4×GGG/*P31 substrate (and 4×GGG oligomer) still preferentially form intramolecular species that preclude formation of intermolecular G-quadruplexes, while the 3×GGG/*P31 substrate and 3×GGG oligomers can only assemble into intermolecular species. Taken together, these results indicate that, under the conditions used for subsequent primer extension assays (75 mM KCl and low DNA substrate concentrations), the single-stranded region of the 4×GGG/*P31 substrate specifically assembles into an intramolecular G-quadruplex while that of the 3×GGG/*P31 substrate remains unfolded. These results also suggest the G-quadruplex detected by DMS protection (Fig. 3B) existed in a unimolecular form.

**Figure 4:**
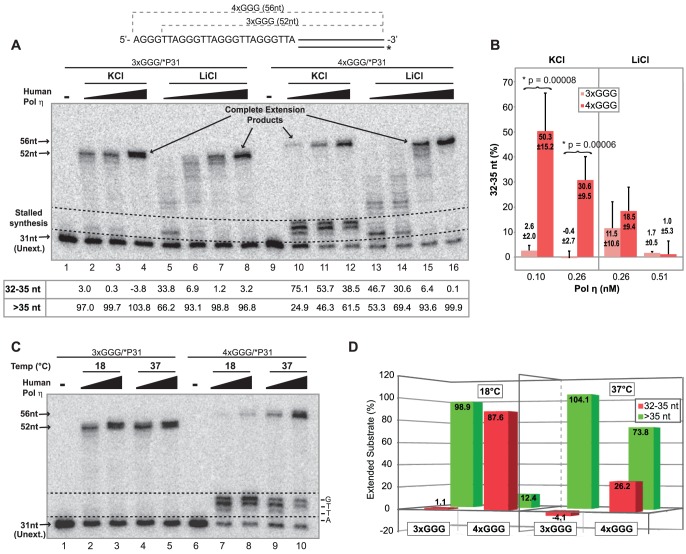
Stability of Intramolecular G-quadruplexes in Template Strand of 4×GGG/*P31. A) Primer extension assays were performed by incubating human pol η (0.05, 0.10, and 0.26 nM in KCl or 0.10, 0.26, 0.51, and 1.0 nM in LiCl) with 3×GGG/*P31 or 4×GGG/*P31 substrate (0.3 nM each) in extension buffer containing 75 mM KCl or LiCl at 37°C for 5 min. Radiolabeled primer strands were separated and visualized after denaturing PAGE. Dotted lines (and in *C* below) delineate the positions of partial extension products resulting from polymerase inhibition by an intramolecular G-quadruplex. Below each lane, quantitation of 32–35 nt and full extension products (>35 nt), as a percentage of only extended products, is shown. B) Bar graph of data collected from multiple independent experiments, performed as described in *(A)*. The percentage of extended products in the 32–35 nt range was plotted at various pol η concentrations; negative values are derived from signal intensities slightly less than the corresponding regions of DNA substrate minus enzyme lanes. Statistical significance (denoted by asterisks) between amounts of 32–35 nt products generated on 4×GGG/*P31 vs. 3×GGG/*P31 was determined by paired T tests. C) To determine the effect of temperature on G-quadruplex stability, primer extension assays were performed at 18°C and 37°C with human pol η (0.26 and 0.51 nM at 18°C or 0.10 and.26 nM at 37°C) on 3×GGG/*P31 or 4×GGG/*P31 (0.3 nM each) in extension buffer with 75 mM KCl for 5 min. As in *A*, percentages of 32–35 nt and >35 nt products are shown below each lane. D) Bar graph showing percentages of 32–35 nt (*red, front row*) and >35 nt (*green, back row*) products from data in *C* at one specific pol η concentration (0.26 nM).

To further examine the secondary structure of the 4×GGG/*P31 template, we also examined the effects of template sequences on DNA synthesis. Using a range of human pol η concentrations, we performed primer extension assays at 37°C on 4×GGG/*P31 and 3×GGG/*P31 substrates in 75 mM KCl or LiCl. Extension of the labeled primer strand (31 nt) was examined by denaturing PAGE (Fig. 4A). As mentioned above, LiCl favors unstructured DNA, while KCl promotes G-quadruplex formation [22]. In KCl, human pol η fully extended the primer strand of 3×GGG/*P31 substrate to primarily generate a 52 nt product (Fig. 4A, lanes 2–4), indicating an unfolded single-stranded template. The extension pattern for 4×GGG/*P31 was drastically different in KCl (lanes 10–12). Specifically, the most prominent bands observed are 34 and 35 nt in length, demonstrating extension of the primer by 3 and 4 nt, respectively. Importantly, these bands correspond to polymerase stalling across from the position of the first GGG run in the template that would be involved in presumed intramolecular G-quadruplex structures. Notably, some full extension (56 nt) was observed with increasing polymerase concentrations. Data from multiple experiments demonstrates that, at two different human pol η concentrations, amounts of the 32–35 nt products (expressed relative to total extended products) representing inhibited DNA synthesis before or at the first GGG run were elevated and highly statistically significant (50.3 and 30.6% with p values of 0.00008 and 0.00006, respectively) on the 4×GGG template as compared to basically undetectable levels (2.6 and −0.4%, respectively) of these products on the control 3×GGG template (Fig. 4B). In contrast, both the 4×GGG/*P31 and control 3×GGG/*P31 substrates showed similar extension profiles and product patterns at equivalent enzyme amounts in LiCl (Fig. 4A, lanes 5–8 and 13–16) with similar levels of complete extension observed at the higher pol η concentrations. Most importantly, the prominent 34 and 35 nt products generated on the 4×GGG/*P31 substrate in KCl are not observed to any sizeable degree in LiCl (Fig. 4A, lanes 10–16, and Fig. 4B). Taken together with our previous DMS and native PAGE analyses, these results further confirm that the single-stranded regions of the template strands of 4×GGG/*P31 folds into intramolecular G-quadruplexes in 75 mM KCl. Notably, assembly of single-stranded G-rich human telomeric DNA sequences containing 4 GGG runs into intramolecular G-quadruplex structures in KCl solutions is in complete agreement with previous reports [26], [27], [28]. In contrast, the template strand of 3×GGG/*P31 substrate containing only three GGG runs remains in an unfolded, single-stranded state in both KCl and LiCl, supporting use of 3×GGG/*P31 and 3×GGG/*P34 substrates as optimal controls for all subsequent primer extension studies.

The presence of both truncated and fully extended products in our primer extension assays performed with pol η on 4×GGG/*P31 substrate at 37°C in 75 mM KCl (Fig. 4A, lanes 10–12) suggested that its template region might exist in dynamic equilibrium between intramolecular G-quadruplex and unfolded states. To better understand formation and stability of these intramolecular G-quadruplexes before continuing our studies with DNA polymerases, we investigated the effects of two parameters known to affect G-quadruplex stability–i.e., temperature and monovalent cation (K^+^) concentration [29]. First, we examined the effect of temperature (18°C vs. 37°C) on human pol η extension of G-quadruplex-forming substrate, 4×GGG/*P31, compared to control substrate, 3×GGG/*P31, in buffer containing 75 mM KCl (Fig. 4C). Human pol η completely extended the control 3×GGG/*P31 substrate at 18°C (lanes 2–3) and 37°C (lanes 4–5) to generate 52 nt products, with temperature having little or no effect on polymerase activity. In contrast, assays with G-quadruplex-forming 4×GGG/*P31 substrate (lanes 6–10) exhibit a markedly different pattern and a substantial temperature effect. At 37°C, 4×GGG/*P31 was extended 1–4 nt (32–35 nt), indicating inhibition of polymerization at an intramolecular G-quadruplex (lanes 9–10). However, at the highest human pol η concentration, a large percentage (54%) of fully extended product (56 nt) was also generated (lane 10). At 18°C, much less full extension was observed at this polymerase concentration (lane 8) along with an increased amount of products correlating with blockage across from the G-quadruplex (lanes 7–8). We quantified the amounts of both 32–35 nt and >35 nt extension products, the former relevant to stalling in the vicinity of the first GGG run in the template and the latter reflecting synthesis beyond this point (Fig. 4C), and graphically compared these products for both substrates at 18°C and 37°C at a fixed polymerase concentration (Fig. 4D). On the intramolecular G-quadruplex-forming 4×GGG/*P31 substrate, a much higher proportion of 32–35 nt products (red bars) is observed at 18°C (87.6%) versus 37°C (26.2%), indicating that stalling of human pol η is much more predominant at the lower temperature. In contrast, little or no difference at 18°C versus 37°C is observed on the control 3×GGG/*P31 substrate for which essentially no stalling occurs and all the products are >35 nt (green bars), with the vast majority of this group being the completely extended, 52 nt products (Fig. 4C, lanes 2–5 and Fig. 4D).

We also compared primer extension of 4×GGG/*P31 and 3×CCC/*P31 at 37°C in 50 mM or 100 mM KCl using Kexo^−^, a more processive polymerase than human pol η. The primer strand of control 3×CCC/*P31 substrate was extended in 50 and 100 mM KCl (Fig. S2A, lanes 1–8), with higher levels of complete extension occurring as Kexo^−^ concentrations increase, although overall Kexo^−^ activity was somewhat reduced at 100 mM KCl. On 4×GGG/*P31 substrate, there was evidence of G-quadruplex-mediated inhibition of Kexo^−^ as well as a substantial effect of KCl concentration (Fig. S2A, lanes 9–16). Quantitation of the 32–35 nt and >35 nt products as above demonstrated little or no stalling on the unfolded 3×CCC/*P31 substrate (Fig. S2A, lanes 2–8). However, on the G-quadruplex forming 4×GGG/*P31 substrate, consistently higher levels of the 32–35 nt products were observed at 100 mM KCl than at 50 mM KCl over the range of Kexo- concentration (Fig. S2A, lanes 10–16). This difference between 50 and 100 mM KCl related to stalling on the 4×GGG template was particularly pronounced at the highest polymerase concentration tested (Fig. S2B). While a very minor part of this decrease in extension at 100 mM KCl may result from reduced polymerase activity, these results strongly support the view that intramolecular G-quadruplexes formed from the single-stranded template region of 4×GGG/*P31 are significantly more favored in 100 mM KCl versus 50 mM KCl. Taken together, these results strongly suggest that the telomeric template region of the 4×GGG/*P31 substrate is in dynamic equilibrium between its unfolded single-stranded state and an intramolecular G-quadruplex structure. Lower temperatures and increased KCl concentrations shift that equilibrium towards G-quadruplex formation and stability. By this reasoning, encounters between polymerase molecules and unfolded templates are much rarer at 18°C than at 37°C (and at higher versus lower KCl concentration), explaining the observed marked reduction in full length products at the lower temperature (and in 100 mM KCl).

### Effects of Intramolecular G-quadruplexes on replicative and translesion DNA polymerases

**Figure 5:**
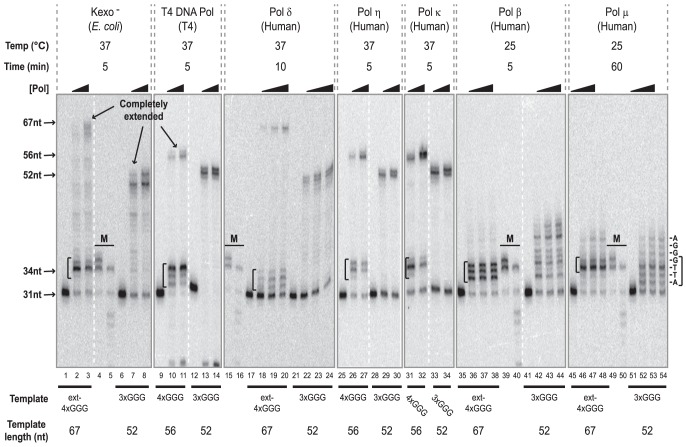
Inhibition of Replicative and Translesion Polymerases by Intramolecular G-quadruplexes. In primer extension assays containing 75(3×GGG/*P31) and G-quadruplex-forming (4×GGG/*P31 or ext-4×GGG/*P31) substrates (0.2–0.3 nM) were incubated with increasing concentrations of the following polymerases: Kexo^−^ (10 and 20 U/L), T4 DNA polymerase (50 and 100 U/L), pol δ (3.4, 8.6, and 17.1 nM), human pol η (0.10 and 0.26 nM), pol κ (1.3 and 2.5 nM), pol μ (5.5, 10.9, and 27.3 nM), or pol β (11.6, 23.2, and 46.3 nM). Incubation temperatures and times for individual polymerases are specified above the gel. Markers (M) generated by 3×GGG/*P31 extension using dATP and dTTP only and Kexo^−^ (lanes 4, 15, 39, and 49) or T4 DNA polymerase (lanes 5, 16, 40, and 50) are denoted. Positions of partial extension products indicating polymerase stalling are denoted with brackets. White dashed lines indicate where separated groups of lanes from a single gel have been spliced together.

The results above demonstrate formation of intramolecular G-quadruplex structures in the template strand of our 4×GGG/*P31 substrate and indicate that these structures appear to inhibit DNA synthesis by human pol η and Kexo^−^. However, organisms possess various DNA polymerases that may respond in different ways to these structures, including polymerases involved in synthesis of long DNA tracts (such as Kexo^−^, T4 DNA polymerase, and human pol δ) or more distributive DNA polymerases (including human pol η, pol κ, pol μ, and pol β) involved in lesion bypass or DNA repair (for more details, see Table S2). Therefore, we wanted to investigate the effect of an intramolecular G-quadruplex in the template on extension by a variety of DNA polymerases. Here, we used the intramolecular G-quadruplex-forming 4×GGG/*P31 substrate with a 56 nt template strand as well as a new substrate, ext-4×GGG/*P31 containing a 67 nt template strand identical to 4×GGG/*P31 except for an 11 nt 5′ extension of random sequence that should assume a single-stranded conformation (Fig. 2), allowing use of this substrate to enable detection of possible “skipping over” of G-quadruplexes by various polymerases. Our experimental strategy was, for each polymerase independently, direct comparison of primer extension on the control 3×GGG/*P31 substrate and a G-quadruplex-forming substrate, either 4×GGG/*P31 or ext-4×-GGG/*P31. For each polymerase we used multiple enzyme concentrations, determined by the minimum amounts needed to achieve either complete extension to the end of the template or, for highly distributive pol μ and pol β, sufficient extension past the initial GGG run in the template of the unfolded control substrate. Reaction conditions, specified in Fig. 5, were similar for all polymerases (5–10 min at 37°C in buffer containing 75 mM KCl) except pol μ and pol β. Quantitation of 32–35 nt and >35 nt products for these reactions was performed, with respect to only the extended products. Table 1 shows values for the short (32–35 nt) products relevant to stalling in the vicinity of the first GGG run in the templates.

**Table 1:** Replicative and Translesion Polymerases are Blocked by Intramolecular G-quadruplexes.

	32–35 nt (%)^a^																
	Kexo^−^		T4		Pol δ			Pol η		Pol κ		Pol β			Pol μ^b^		
	[Low]	[High]	[Low]	[High]	[Low]	[Med]	[High]	[Low]	[High]	[Low]	[High]	[Low]	[Med]	[High]	[Low]	[Med]	[High]
3×GGG	8.2	4.8	−0.8^c^	−0.2	1.9	−1.5	−1.1	4.5	−1.3	2.2	0.9	43.5	28.1	23.8	39.2	42.3	41.7
4×GGG	57.8	32.3	82.5	72.1	75.0	61.7	56.1	63.1	34.1	55.8	23.1	81.9	81.1	81.1	69.5	64.6	64.6

^a^ Each value represents the percentage of products 32–35 nt in length, with respect to the total amount of extended products.

^b^ For pol μ, only the sum of the 34 and 35 nt products, compared to the total amount of extended products, is presented.

^c^ Negative values throughout represent instances in which the signal intensity in the 32–35 nt range for lane containing enzyme was not greater than the intensity in that respective region for the lane containing only DNA substrate.

As expected from our earlier experiments, Kexo^−^ extends the control template, 3×GGG/*P31, to completion (52 nt) in a manner related to enzyme concentration (Fig. 5, lanes 6–8). In contrast, when ext-4×GGG/*P31 substrate was utilized (lanes 1–3), the substantial levels of 32–35 nt products that includes the prominent 34 and 35 nt bands indicate inhibition of Kexo^−^ by intramolecular G-quadruplexes, even though low amounts of longer (>35 nt, up to the fully extended 67 nt) products are observed, with their relative levels correlating to polymerase concentration (Table 1). When the replicative T4 DNA polymerase was employed, results were generally similar even though slightly different product patterns were observed. Although some products even shorter than the primer were detectable, reflecting degradation by this enzyme's inherent 3′ to 5′ exonuclease activity [30], we mainly observed full extension of the primer of the 3×GGG/*P31 substrate (lanes 12–14), with little or no 32–35 nt products (Table 1; 8.2 and 4.8%, at the low and high enzyme concentrations, respectively) and almost all (91.8 and 95.2%, respectively) of the extended products being >35 nt. However, with the G-quadruplex-forming 4×GGG/*P31 substrate (lanes 9–11), the primary products (82.5 and 72.1%, at the low and high enzyme concentrations, respectively) observed with T4 DNA polymerase were between 32–35 nt in length (Table 1) with minor amounts of fully extended (56 nt) products. Similar to T4 DNA polymerase, human pol δ, the main lagging strand replicative DNA polymerase [31], fully extends the control substrate (lanes 21–24) and generates primarily 32–35 nt products (75.0, 61.7, and 56.1% at the three concentrations employed) using the ext-4×GGG/*P31 substrate (lanes 17–20) with some full extension (67 nt) occurring as polymerase concentrations increase.

Close visual inspection of the product patterns generated on the intramolecular G-quadruplex-forming templates indicates that products of 34 and 35 nt are primarily generated by Kexo- (and most translesion polymerases, see below), compared to products between 32–34 nt observed with T4 DNA polymerase and human pol δ, both of which possess 3′ to 5′ exonuclease (proofreading) activity. To further investigate possible reasons for these slightly shorter product distributions, we directly compared the actions of exonuclease-proficient and -deficient T4 DNA polymerase on control and G-quadruplex-forming substrates (Fig. S3). Reactions on the G-quadruplex-forming (4×GGG/*P31) substrate, performed over concentration ranges reflecting comparable synthetic activity of these enzymes, again revealed products predominantly between 32–34 nt with both enzymes (Fig. S3, lanes 2–7). Compared to exonuclease-deficient T4 polymerase, the exonuclease-proficient enzyme yielded modestly higher levels of 32 and 33 nt products, likely reflecting some proofreading of the 34 nt species (compare lanes 2–4 with lanes 5–7); this 3′ to 5′ exonuclease activity appears quite robust, as judged by the pattern of products generated on the control substrate (Fig. S3, lanes 9–11). Most intriguingly, this 32–34 nt product distribution and the absence of a pronounced 35 nt product, particularly using the exonuclease-deficient enzyme, suggests that intramolecular G-quadruplexes inhibit synthesis by T4 DNA polymerase slightly further upstream than for Kexo- and translesion polymerases. It is tempting to speculate that the same paradigm may apply for human pol δ, also a replicative polymerase that generates 32–34 nt products on the G-quadruplex-forming template.

Next, we examined the human translesion polymerases pol η and pol κ. As anticipated from earlier results, human pol η extends the control 3×GGG/*P31 substrate (Fig. 5, lanes 28–30) to completion (52 nt), but on the G-quadruplex-forming 4×GGG/*P31 substrate generates mainly 34 and 35 nt products, corresponding to stalling at the site of the first GGG run, as well as some completely extended (56 nt) products (lanes 25–27). Quantitation of this data indicates that, on the ext-4×GGG/*P31 substrate, 63.1 and 34.1% of the extended products are 32–35 nt in length at the low and high pol η concentrations, respectively; in contrast, little or no products of this length are detected on the control 3×GGG/*P31 substrate (Table 1), with essentially all of the extended products being >35 nt. Human pol κ shows extension patterns very similar to human pol η–i.e., it fully extends the control substrate (lanes 33–34) and a substantial fraction of the 4×GGG/*P31 substrate (lanes 31–32) but is also frequently stalled by G-quadruplex formation on the latter, as evidenced by the prominent 33–35 nt bands. Consistent with this observation, on the 4×GGG/*P31 substrate 55.8 and 23.1% of the extended products are 32–35 nt at the low and high pol κ concentrations, while little or no (<2.2%) products of this length are generated on the 3×GGG/*P31 substrate (Table 1). These results demonstrate that synthesis by both human pol η and pol κ is dramatically inhibited by the presence of an intramolecular G-quadruplex in the template. Notably, synthesis by *S. cerevisiae* pol η is also inhibited by the presence of intramolecular G-quadruplexes (Fig. S4); however, the lower physiological temperature for this enzyme also impacts G-quadruplex stability, making direct comparisons with the other polymerases tested here difficult.

We also tested the effect of intramolecular G-quadruplexes on two very distributive X family DNA polymerases: 1) human pol β that acts in base excision repair and 2) human pol μ, an error-prone polymerase that can bypass DNA lesions and may act in non-homologous end joining and VDJ recombination [32]. Because of the highly distributive nature of these polymerases, higher enzyme concentrations and, for pol μ, longer incubation times (up to 60 min) were required to achieve sufficient synthesis past the first GGG run on the control template. However, these conditions also increase the probability that the polymerase would encounter G-quadruplex-forming substrates when the template was in an unfolded state, particularly at 37°C for which we have demonstrated the relatively dynamic nature of these structures (Fig. 4C and D). Therefore, experiments using pol β and pol μ were performed at lower temperatures (25°C for Fig. 5) to further stabilize intramolecular G-quadruplexes. Unlike the previous polymerases tested, pol β extended the control substrate by only 1–11 nt to yield products in the range from 32–42 nt (Fig. 5, lanes 42–44), confirming the very distributive nature of this polymerase even on an unfolded and undamaged template. Nevertheless, on the G-quadruplex-forming ext-4×GGG/*P31 substrate, the distribution of extended products was clearly shorter in length, with the vast majority of the extended products being 33–35 nt in length, again corresponding closely to the position of the first GGG run in the template (Fig. 5, lanes 36–38). As before, we quantified the levels of the 32–35 nt and >35 nt products in these reactions, even though the distributive nature of pol β results in substantial contributions to the short 32–35 nt products on the control substrate. Despite this limitation, substantially higher levels of these 32–35 nt products were generated on the G-quadruplex-forming substrate than on the control–i.e., 81.9, 81.1, and 81.1% compared to 43.5, 28.1 and 23.8% at the low, medium and high concentrations, respectively (Table 1). These results indicate that synthesis by human pol β is also inhibited by the presence of intramolecular G-quadruplexes. Our findings with human pol μ were less clear than for other polymerases, primarily because we had difficulty getting sufficient synthesis beyond the first GGG run on the control substrate even over 60 min. Specifically, pol μ only extends the control 3×GGG/*P31 substrate (Fig. 5, lanes 52–54) between 1–8 nt (32–39 nt), with a modest pause site at 34 nt. However, on the ext-4×GGG/*P31 substrate, pol μ generates a different pattern, with enhanced pause sites at 34 and 35 nt reflecting increased stalling after adding 3–4 nt, again correlating to the position of putative G-quadruplexes in the template (lanes 46–48). Calculation of the extended products (Table 1) indicated much higher amounts of these 34 and 35 nt products when using the G-quadruplex-forming substrate (ranging between 64.6–69.5%) compared to the control substrate (between 39.2–42.3%). We attribute products longer than 35 nt on the ext-4×GGG/*P31 substrate to some dynamic unfolding of the G-quadruplex over the extended 60 min incubation interval. These results suggest human pol μ is also inhibited to some extent by the presence of an intramolecular G-quadruplex in the template.

In summarizing the results depicted in Fig. 5, we examined the activity of several DNA polymerases under conditions (75 mM KCl) in which the template strands of the 4×GGG/*P31 test substrate have been demonstrated (see Figs. 3,4) to fold into intramolecular G-quadruplex structures. Importantly, synthesis by all polymerases examined in this study was stalled prior to or at the first GGG run in the template–i.e., precisely corresponding to the 3′ most sequence that would be involved in intramolecular G-quadruplex formation for 4×GGG/*P31 and ext-4×GGG/*P31 substrates. Notably, enzymes lacking exonuclease activity, including Kexo^−^, human pol η, *S. cerevisiae* pol η, human pol κ, human pol μ, and human pol β, generate not only 34 nt products reflecting synthesis up to the first GGG run in the G-quadruplex-forming template but also significant amounts of 35 nt product. The lack of a 35 nt product as well as the prominence of 32–33 nt products in reactions with T4 DNA polymerase and pol δ (compared to reactions with Kexo- and translesion polymerases) may be due to stalling further upstream of this structure, although their proofreading activities may contribute to the prevalence of shorter products. Nevertheless, these results demonstrate that a wide range of polymerases, including translesion polymerases that bypass bulky DNA lesions, are markedly inhibited upon encountering intramolecular G-quadruplexes in the template strand.

### Translesion Polymerases May Promote Mutagenesis at G-quadruplexes

Previous results show the 4×GGG/*P31 and ext-4×GGG/*P31 templates form intramolecular G-quadruplexes (in 75 mM KCl) that inhibit synthesis by human pol η and other DNA polymerases (Figs. 4–5). Although these secondary structures dramatically inhibited extension by all DNA polymerases tested here, many of our primer extension assays on G-quadruplex-forming substrates (particularly those employing translesion polymerases) revealed significant levels of 35 nt products that would, under normal circumstances, be specified by the initial guanine in the first GGG run on the template strand. Since this guanine might be involved in the intramolecular G-quadruplex structure that inhibits synthesis, generation of this 35 nt product raises the possibility that an intramolecular G-quadruplex in the template might lead to misincorporation that could subsequently result in mutagenesis. To address this possibility, we initially assessed the fidelity of incorporation by pol η by limiting nucleotide availability in assays with the 3×GGG/*P31 and ext-4×GGG/*P31 substrates used above, since only dATP and dTTP are required to extend their primer strands up to the initial run of guanines (see Fig. 4A, top). These primer-extension assays were performed in 75 mM KCl and at 18°C, conditions that further stabilize G-quadruplexes, in order that outcomes can be more directly attributed to the presence of an intramolecular G-quadruplex in the template. Our results showed that pol η generated substantial amounts of the 35 nt product in reactions performed with control (unfolded) 3×GGG/*P31 substrate, indicating misincorporation of either dATP or dTTP (Fig. S5, lanes 5–7). On the G-quadruplex-forming ext-4×GGG/*P31 substrate, lower but clearly detectable levels of the 35 nt products were observed in reactions with only dATP and dTTP (Fig. S5, lanes 12–14), revealing that pol η can potentially incorporate an incorrect nucleotide under conditions in which the template contains an intramolecular G-quadruplex.

**Figure 6:**
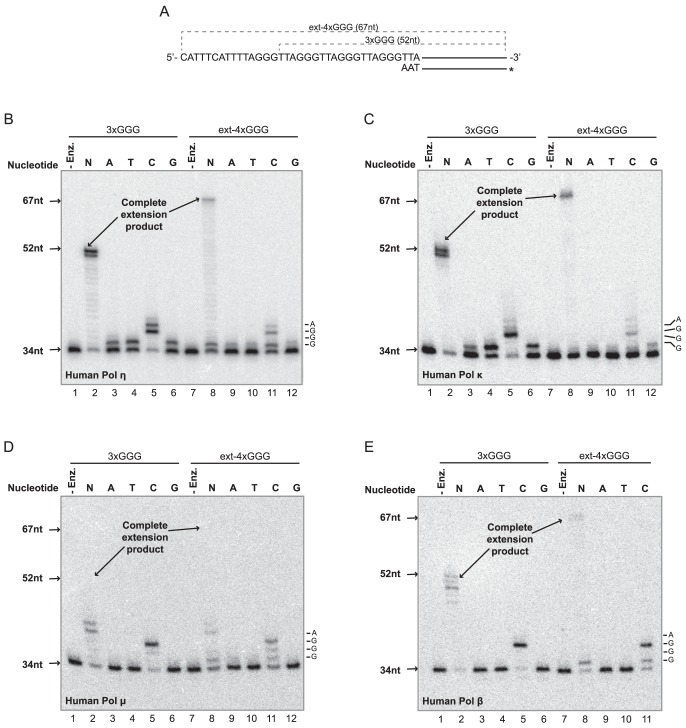
Nucleotide Misincorporation by Translesion and Repair DNA Polymerases on Unfolded and Intramolecular G-quadruplex-containing Substrates. A) Structure and template sequences of 3×GGG/*P34 and ext-4×GGG/*P34 substrates. B–E) In the presence of either all dNTPs (N) or dATP (A), dTTP (T), dCTP,(C) or dGTP (G) individually (100 µM each), primer extension assays were performed on 3×GGG/*P34 or ext-4×GGG/*P34 (0.4 nM) in extension buffer with 75 mM KCl using *B)* human pol η (1.0 nM) at 18°C for 5 min, *C)* human pol κ (0.84 nM) at 18°C for 5 min, *D)* human pol μ (27.3 nM) in 10% glycerol at 25°C for 60 min, and *E)* human pol β (28.9 nM) at 18°C for 30 min. The first four nucleotides in the template are depicted at right at positions correlating to their respective primer products.

To more closely examine incorporation when an intramolecular G-quadruplex is present in the template, we created substrates with the same template strands but using longer (34 nt) primer strands. The 3′ ends of these primers abut the initial guanine of the first GGG run in the template strands of the control 3×GGG/*P34 substrate and the intramolecular G-quadruplex-forming ext-4×GGG/*P34 substrate (Fig. 6A). We then assayed primer extension at 18°C in 75 mM KCl using all dNTPs or dATP, dTTP, dCTP, or dGTP individually; in these assays, we focused primarily on the error-prone human translesion polymerases (pol η, pol μ and pol κ) that are thought to be recruited in response to replication-blocking events and also lack proofreading activity. Importantly, reactions performed with all dNTPs on control and G-quadruplex forming substrates (Fig. 6B–E, lanes 2 and 8 in each panel) confirmed our conclusion that intramolecular G-quadruplexes pose a substantial obstacle to DNA synthesis by these polymerases. Specifically, for each individual polymerase, visual inspection consistently shows more unextended 34 nt primers in reactions performed with G-quadruplex-forming compared to control substrates. However, this point is best illustrated by quantifying together the amounts of 34 and 35 nt species that reflect no extension and incorporation of one nucleotide, respectively, keeping in mind that these products directly parallel the sites of inhibition observed with substrates containing shorter (31 nt) primers (see Figs. 4–5). This analysis (Table S3) demonstrates that much greater amounts of the reaction products (60.7, 54.9, 56.3 and 42.5% for human pol η, pol κ, pol μ and pol β, respectively) were 34–35 nt on the G-quadruplex-forming substrates as compared to the control substrates (15.5, 18.5, 27.4 and 16.7%, respectively), on which pol η and pol κ mostly generated products reflecting complete extension. Notably, these results with human pol μ (Fig. 6D, compare lanes 2 and 8) reinforce our earlier interpretation that this highly distributive translesion polymerase is substantially inhibited by an intramolecular G-quadruplex. Thus, the results from dNTP-containing reactions on these substrates with longer (34 nt) primers are in complete agreement with our previous findings (Fig. 5) regarding the inhibitory effects of intramolecular G-quadruplexes in the template on synthesis by these polymerases and the position at which this inhibition occurs.

Examination of results obtained with individual nucleotides were also revealing. Reactions on the unfolded control templates containing only dATP, dTTP, or dGTP (Fig. 6B–E, lanes 3, 4, and 6 in each panel) show substantial levels of single nucleotide misincorporation at the first GGG run in the template to yield 35 nt products when either pol η or pol κ is employed (Fig. 6B,C; Table S4), consistent with their previously reported low fidelity on undamaged templates [33], [34]. For this sequence context, pol κ preferentially misincorporated dTTP (58.6%) over dGTP (28.6%) and dATP (17.4%), while pol η misincorporated each at a more similar frequency with perhaps a modest preference for dTTP (42.6, 30.0 and 28.2%, respectively). On this control substrate, misincorporation of dTTP, dGTP, or dATP by pol μ was below the minimum detection limit even though very faint 35 nt bands can be seen in dTTP and dGTP lanes (Fig. 6D; Table S4). No misincorporation by pol β was detected (Fig. 6E; Table S4), in agreement with its role as a repair polymerase with relatively higher fidelity than translesion polymerases. As expected, incorporation of multiple dCTP residues opposite the initial GGG run in the unfolded template was readily observed with all these polymerases (Fig. 6B–E, lane 5 in each panel). While pol β and pol μ primarily incorporated three cytosines to predominantly yield 37 nt bands with minor amounts of 38 nt products, pol η and pol κ generated substantial amounts of 37 and 38 nt products and even readily detectable amounts of 39 nt products (Table S5). These 38 and 39 nt products could potentially be generated by two mechanisms: 1) misincorporation of dCTP opposite the adenine and thymine at the fourth and fifth positions in the template or 2) slippage of the primer strand at the GGG run, leading to additional dCTP incorporation events specified by guanine in the template. The presence of detectable levels of 39 nt products with pol η and pol κ would seem to suggest strand slippage on an unfolded template as the favored mechanism for incorporation of these additional cytosines.

Reactions performed on the G-quadruplex-forming ext-4×GGG/*P34 substrates with individual nucleotides were equally informative (Fig. 6B–D, lanes 9–12). Incorporation events on these substrates were generally less than for control substrates, almost assuredly because of the previously documented inhibitory effects of intramolecular G-quadruplexes on synthesis by these polymerases. In reactions containing dATP, dTTP, or dGTP and the G-quadruplex-forming substrate, translesion polymerases occasionally added a single nucleotide to the 34 nt primer to generate 35 nt products (see frequencies in Table S4), but longer products were not observed. With pol η (Fig. 6B), the levels of misincorporation (with respect to the total products for each reaction) of dATP (lane 9), dTTP (lane 10), or dGTP (lane 12) were 9.0%, 7.1%, and 5.5%, respectively. Similar levels of misincorporation of dATP (6.6%), dTTP (4.4%) and dGTP (8.5%) were observed with pol κ (Fig. 6C). When pol μ (Fig. 6D) was used, some misincorporation of dATP (7.7%) and dTTP (3.6%) was observed, while no addition of dGTP was detected. There was little or no detectable misincorporation by pol β on the G-quadruplex-containing substrate (Fig. 6E), similar to results with the control substrate. Curiously, the preference of pol κ to misincorporate dTTP (over either dATP or dGTP) observed on control substrate is not apparent on the G-quadruplex-containing substrate (Table S4). Nevertheless, our results suggest that pol η, pol κ, and pol μ can potentially catalyze misincorporation when an intramolecular G-quadruplex is present in the template. More complicated patterns of products were observed on this G-quadruplex-containing substrate with dCTP alone (Fig. 6B–E, lane 11 in each panel). Extension by all polymerases in the presence of dCTP occurred with greater frequency, as evidenced by a reduced amount of unextended 34 nt primers compared to reactions containing dATP, dTTP, or dGTP (Table S4). Correspondingly, products between 35–39 nt are observed (Table S5), reflecting incorporation of 1–5 cytosines. Products longer than 35 nt would appear to stem from templates that assume an unfolded state before or during the course of the reaction. We attribute the 37 nt product to correct incorporation of three cytosines opposite the initial GGG run. However, the 38 and 39 nt products generated more often with pol η and pol κ (than with pol μ and pol β) are likely explained by the strand slippage mechanism mentioned above regarding results using the control substrate. Exact interpretation of the 35 nt product (and its relative prominence) in reactions containing pol η, pol κ, and pol β with only dCTP is more challenging. However, its greater abundance compared to consistently lower levels of 36 nt product suggests that the 35 nt product is directly attributable to the presence of intramolecular G quadruplexes in the template. Since the frequency of these 35 nt products is higher with all polymerases tested with dCTP than for dATP, dTTP, or dGTP (Table S4), these results strongly suggest that these polymerases may have some preference for incorporation of dCTP at this particular position. This was also observed with yeast pol η (data not shown). Importantly, any preference for dCTP in these reactions may even be underestimated, as its incorporation may tend to de-stabilize G-quadruplex structures and thereby lead to further extension (and increased levels of products longer than 35 nt). Taken together, these results at least suggest preferential but not exclusive incorporation of cytosine when an intramolecular G-quadruplex was present in the template. Misincorporation of adenine, thymine or guanine was observed with pol η, κ and μ. Regarding the design of these experiments, we also reasoned that the additional 11 nt present on the template strand of ext-4×GGG/*P34 might facilitate occurrence and observation of possible “skipping over” of intramolecular G-quadruplexes by translesion polymerases, as previously observed for pol μ on templates containing DNA lesions [35]. However, we did not detect any prominent products in the 40–50 nt range that might result from skipping over the G-quadruplex by human pol η or other translesion polymerases tested (Fig. 6B–E, lane 8 in each panel).

**Figure 7:**
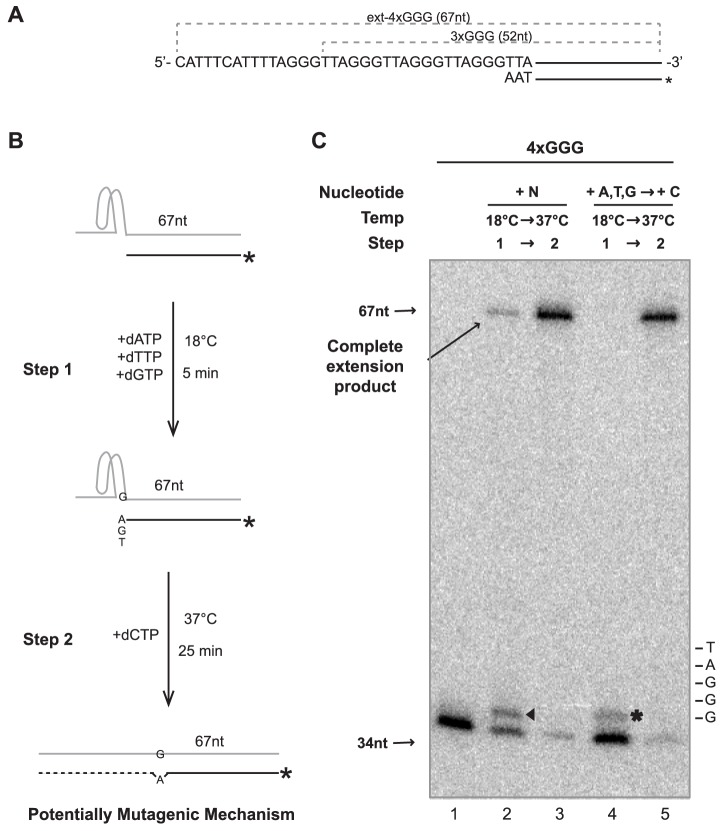
Intramolecular G-quadruplex-induced Misincorporation and Extension by Human Pol η. A) Structure and sequence of ext-4×GGG/*P34 substrate. B) Protocol for determining ability of human pol η to promote mutagenesis when encountering an intramolecular G-quadruplex. C) A primer extension assay was performed in two steps. Initially, 4×GGG/*P34 (0.4 nM) was incubated with human pol η (1.0 nM) with all dNTPs (N), dATP (A), dTTP (T), or dGTP (G) (100 µM each) at 18°C for 5 min. Upon completion of step 1, the reaction was supplemented with 100 µM dCTP (C) and incubated at 37°C for 25 min. Aliquots (4 µL) of the reaction were removed immediately after completion of each step for analysis by denaturing PAGE. The asterisk (lane 4) highlights products generated due to misincorporation putatively across from the first guanine that could be involved in an intramolecular G-quadruplex. The arrowhead highlights products generated by incorporation by human pol η upon encountering the intramolecular G-quadruplex in the presence of all dNTPs.

Misincorporation by pol η or another DNA polymerase followed by continued extension by the same or a different polymerase may lead to mutagenesis. To investigate possible promotion of mutagenesis at G-quadruplexes, we developed a protocol (Fig. 7B) to determine if human pol η could extend from a nucleotide misincorporated due to encountering an intramolecular G-quadruplex in the template. In step 1, we promoted misincorporation of a single nucleotide opposite a stable intramolecular G-quadruplex by incubation of 4×GGG/*P34 (Fig. 7A) with dATP, dTTP, and dGTP and human pol η at 18°C, a temperature that stabilizes G-quadruplexes but still permits synthesis by pol η. Then, in step 2, we encouraged further extension to occur by simultaneously adding dCTP and increasing the temperature to 37°C to somewhat destabilize G-quadruplexes (Fig. 7B). As a separate control, each step was performed in the presence of all dNTPs. Aliquots of these reactions were removed following each step for analysis of extension of the primer strand by denaturing PAGE (Fig. 7C). When parallel experiments were performed on the unfolded control 3×GGG/*P34 substrate, human pol η both inserted a misincorporated nucleotide in the absence of dCTP and extended from the 3′ mismatch following Step 2, a phenomenon previously reported [36]; as expected, full extension occurred when all dNTPs were available (data not shown). When the G-quadruplex forming ext-4×GGG/*P34 substrate was employed while limiting the available nucleotides to dATP, dTTP, and dGTP, misincorporation was observed in step 1 to generate 35 nt products (Fig. 7C, lane 4, denoted by asterisk). After raising the temperature and addition of dCTP (step 2), this 35 nt product disappears completely (along with most of the remainder of the original substrate) concomitant with appearance of fully extended (67 nt) product (Fig. 7C, lane 5). Thus, under these conditions that enhance unfolding of G-quadruplexes, the specific disappearance of the 35 nt species and its conversion to a fully extended product indicates that human pol η extends those singly misincorporated products that form 3′ mismatches with template. This experiment reveals a scenario by which pol η both misincorporates a nucleotide upon encountering a semi-stable G-quadruplex in the template and subsequently extends from this misincorporated nucleotide upon dissolution of this secondary structure. These results suggest the possibility of mutagenic events at sites where the dynamic folding and unfolding of intramolecular G-quadruplexes alternately stall and permit, respectively, DNA replication by pol η and perhaps other error-prone translesion polymerases (such as pol κ) that might be recruited and employed after replicative DNA synthesis has been initially blocked by these secondary structures.

## Discussion

Numerous G-rich regions of the genome, including long stretches of the telomeric repeat sequence TTAGGG found at both ends of each human chromosome, have the potential to form G-quadruplex structures [11]. These structures are potentially assembled from single-stranded G-rich regions either present on telomeric 3′ overhangs or exposed during DNA replication, with intramolecular G-quadruplex formation presumably being favored in these situations [1], [14]. Importantly, G-rich telomeric repeat sequences are specifically subject to lagging strand replication, likely increasing the propensity for formation of these secondary structures that may impact DNA polymerase function and/or fidelity. It is notable that telomeres resemble fragile sites indicating frequent replication stress [15] that may result, at least in part, from formation of G-quadruplexes. Here, we designed telomeric substrates with the potential to form G-quadruplex structures and examined their effects on DNA synthesis *in vitro* using various DNA polymerases. At the limiting DNA concentrations used in our assays, the template strands of our primer-template substrates (4×GGG/*P31, 4×GGG/*P34, ext-4×GGG/*P31, and ext-4×GGG/*P34) possessing four guanine runs were confirmed to exclusively form intramolecular G-quadruplexes, and we determined conditions that favor G-quadruplex formation and stability. DNA synthesis by a range of polymerases, including those acting in replication and repair (Kexo^−^, T4 DNA polymerase, human pol δ and pol β) and in lesion bypass (*S. cerevisiae* pol η and human pol η, pol κ and pol μ), was dramatically inhibited by intramolecular G-quadruplexes on the template strands (Fig. 4–6, Table 1, Figs. S2, S3, S4 and Table S3). This inhibition of DNA synthesis always occurred proximal to the site where putative G-quadruplexes would be formed in the template, although our results suggest that T4 DNA polymerase and perhaps other replicative polymerases are stalled slightly further upstream of G-quadruplexes than are repair or translesion polymerases. Further analysis of human pol η (as well as human pol κ, and human pol μ) revealed the potential for misincorporation and mutagenesis when intramolecular G-quadruplexes were present in the template. Although intramolecular G-quadruplexes formed specifically from telomeric G-rich sequence were used in our studies, the effects of these structures on replicative and translesion DNA polymerases may be relevant to G-quadruplex-forming sequences throughout the genome. To our knowledge, these results comprise the most comprehensive analysis of the effects of intramolecular G-quadruplexes on DNA synthesis and its fidelity.

Theoretically, our primer-template substrates with single-stranded templates containing human GGGTTA telomeric repeats including 3 or 4 GGG runs have the potential to form intermolecular G-quadruplexes, while only those with 4 GGG runs can form intramolecular G-quadruplexes. We performed a series of experiments including DMS protection analysis, native PAGE migration, and primer extension assays in KCl compared to LiCl to probe for secondary structures formed by our telomeric template sequences. Importantly, at the low DNA concentrations used for our assays, substrates containing only 3 telomeric GGG runs did not detectably form intermolecular G-quadruplexes or other secondary structures, allowing their use as comparative controls in primer extension assays. In contrast, our findings (see Figs. 3,4) conclusively demonstrated that telomeric single-stranded templates containing 4 GGG runs specifically assembled into intramolecular G-quadruplexes, with no detectable formation of intermolecular species at the relatively low DNA concentrations used here. Notably, previous structural analyses of substrates containing G-rich human telomeric sequences indicate that three different intramolecular G-quadruplex structures can be formed in KCl solutions, two of which contain three G-quartets while the other contains only two [26], [27], [28]. Thus, it seems probable that our template sequences assume one or more of these intramolecular conformations. However, since the sequence contexts surrounding our telomeric GGG runs differ sllightly from those used in these structural studies, the precise structures formed (and their relative proportions) are unknown. Our results also indicate that a dynamic equilibrium exists between unfolded DNA and intramolecular G-quadruplex structures formed from human G-rich telomeric repeats, with lower temperatures and higher KCl concentrations favoring G-quadruplex formation and stability (Fig. 4C–D and Fig. S2), in accord with previous findings related to other G-quadruplex structures [29]. Dynamic folding and unfolding of intramolecular G-quadruplexes in genomic DNA, the rates of which would vary depending upon the specific sequences involved, would certainly impact the occurrence of perturbation and/or blockage of replication fork progression and thus the possibilities for 1) recruitment of error-prone polymerases that could promote misincorporation and mutagenesis at these sites or 2) chromosome breakage resulting from replication fork collapse (see below).

Human cells possess many DNA polymerases, including not only polymerases used in DNA replication and repair but also translesion polymerases capable of synthesizing past large, bulky DNA lesions. Using primer extension assays, we found a wide variety of DNA polymerases are stalled by a telomeric intramolecular G-quadruplex on the template strand (Fig. 5). The relatively processive polymerases Kexo^−^, T4 polymerase, and human pol δ that primarily act on undamaged templates, were all inhibited substantially upon encountering an intramolecular G-quadruplex in the template. However, intramolecular G-quadruplexes might be somewhat comparable to sites of bulky DNA damage that frequently block synthesis by processive polymerases. Importantly, distributive translesion polymerases are recruited to sites of replication blockage and employed to replicate past bulky DNA lesions in the template. However, all of the translesion polymerases tested in this study (including *S. cerevisiae* pol η and human pol η, pol κ, and pol μ) were also stalled in very close proximity to the template site at which putative intramolecular G-quadruplexes would be formed with the involvement of template guanine bases. Human pol β, a distributive polymerase involved in base excision repair, was also dramatically inhibited by the presence of intramolecular G-quadruplex structures. In considering the specific effect of intramolecular G-quadruplexes on DNA polymerases, it is worthwhile to compare our primer extension assays performed at physiological temperatures (37°C) versus those performed at lower temperatures (18°C and 25°C) that promote and stabilize G-quadruplex formation (Figs. 4–6). Assays with intramolecular G-quadruplex-forming templates performed at 37°C using any of the tested polymerases (except the highly distributive pol β and μ) show primarily two groups of extended products: 1) DNA species of approximately 56 or 67 nt reflecting complete extension to the ends of the unfolded template strands of 4×GGG and ext-4×GGG, respectively, and 2) short 32–35 nt species corresponding to stalling proximal to the position of putative intramolecular G-quadruplexes in the template. This observation suggests that, while a substantial proportion of the templates retain intramolecular G-quadruplex structures at 37°C, other templates are unfolded or become so during the course of the reaction and are subject to complete extension if encountered by a polymerase molecule. At the lower temperatures that favor G-quadruplex formation and stability, the amounts of full length extension products are consistently reduced while stalling opposite the point of G-quadruplex formation is substantially increased with all polymerases tested. Notably, longer incubation times and/or high polymerase concentrations (for example, in pol μ-containing reactions) allow more opportunities for encounters between polymerase molecules and templates assuming an unfolded conformation, probably explaining generation of products longer than 35 nt in certain reactions. Taken together, we conclude that stable intramolecular G-quadruplexes in the template are formidable obstacles and probably absolute blocks to synthesis by all polymerases tested in our study. Although studies have shown that DNA synthesis by human polymerase δ is inhibited by the presence of multi-stranded intermolecular G-quadruplexes [17], to our knowledge this study is the first to show that human DNA polymerases are blocked by stable intramolecular G-quadruplexes formed from human telomeric sequences. However, comparable findings have been reported using non-human telomeric sequences, non-human polymerases, and/or G-quadruplex-stabilizing ligands [37], [38], [39]. Furthermore, our results demonstrate that these intramolecular structures not only block replicative DNA polymerases but also several translesion polymerases specifically evolved to bypass obstacles to replication. Our results imply that structural perturbations caused by intramolecular G-quadruplexes are too severe for even promiscuous translesion polymerases to overcome. However, we cannot rule out the possibility that some polymerases (or associated proteins) might influence G-quadruplex stability or alter the equilibrium between folded and unfolded states.

Since replicative, repair, and bypass polymerases were all dramatically inhibited in our studies, we believe that DNA polymerases, without aid from other proteins, cannot replicate through or bypass intramolecular G-quadruplexes. Thus, the ability of G-quadruplexes to block both replicative and translesion DNA polymerases may further exacerbate replication difficulties and result in replication collapse and double-strand break formation, consistent with the notion that telomeres resemble fragile sites [15]. Discontinuous lagging strand synthesis generates regions of single-stranded DNA, increasing the potential for intramolecular G-quadruplex formation when this strand is guanine-rich, as is always the case for replication through telomeric DNA. Upon encountering an intramolecular G-quadruplex on the lagging strand, our results indicate human pol δ, the primary polymerase for lagging strand synthesis, would be blocked resulting in incomplete synthesis of the Okazaki fragment. It should be noted that pol δ processivity *in vivo* is substantially increased by PCNA, which was not included in our assays. Although its potential influence cannot be ruled out, PCNA is not believed to help replicative polymerases synthesize past obstructions in DNA; instead, PCNA monoubiquitination upon replication stalling is thought to facilitate recruitment of translesion polymerases [40], [41]. Our results also suggest that recruitment of translesion polymerases such as pol η or pol κ to these sites would be ineffective for bypassing G-quadruplexes. Although downstream leading and lagging strand synthesis likely continues, polymerase blockage by G-quadruplexes formed in telomeric and other genomic regions could generate persistent single-strand gaps eventually leading to double-strand breaks, a hallmark of DNA fragility. Notably, studies in yeast have indicated that non-telomeric regions containing sequence motifs capable of forming G-quadruplexes are also prone to breakage resulting in chromosomal instability [26], [42]. On the other hand, evidence exists in vertebrates that the translesion polymerases REV1, pol η and pol κ have roles in dealing with G-quadruplex structures during replication [16], [38]. A possible scenario, consistent with evidence on REV1 function as well as our results, would suggest that REV1 might be required to assist replication through regions containing G-quadruplexes by translesion polymerases such as pol η and pol κ.

Although our results indicate that an intramolecular G-quadruplex presents a strong barrier to continuous DNA synthesis, we considered the possibility that, as has been observed for specific DNA lesions that impede replicative DNA polymerases, this structure in the template may cause nucleotide misincorporation, potentially leading to mutagenesis. Consistent with this hypothesis, analysis of a range of polymerases revealed that those lacking proofreading (3′ to 5′ exonuclease) activity, including Kexo^−^, human pol η, pol κ, pol μ, and pol β frequently inserted an additional nucleotide onto the primer at a position corresponding to the first guanine that might putatively be involved in intramolecular G-quadruplex structures, generating 35 nt products (Figs. 4–6). To examine these events more closely, we performed primer extension assays upon templates containing a stabilized intramolecular G-quadruplex in the presence of limited or individual nucleotides (Fig. 6 and Fig. S5). These experiments confirmed that these polymerases could incorporate an additional nucleotide even when it seemed likely that the template guanine was involved in the G-quadruplex structure. While putative misincorporation of dATP, dTTP, and dGTP at this position was shown with human pol η, pol μ and pol κ, we intriguingly observed a particular preference for incorporation of dCTP with human pol η and pol μ. Possible explanations for these 35 nt products are: 1) strand slippage events in which the primer realigns with available nucleotides not involved in the G-quadruplex structure that code for standard complementary incorporation, 2) incorporation events occurring without the benefit of a template nucleotide, the template guanine being involved in G-quadruplex structures, or 3) incorporation events mediated by a partially or wholly accessible guanine nucleotide in the template. The first mechanism seems unlikely, since strand slippage events around this position would seem to favor incorporation of dATP, but our results indicate that dTTP or dGTP seem to be incorporated as frequently as dATP. In support of the second mechanism, incorporation of untemplated nucleotides has been observed with Klenow and some translesion polymerases including pol μ [43] [32]. However, the preferential incorporation of dCTP by some polymerases supports the concept that the template guanine might be available, at least in some proportion of the intramolecular G-quadruplexes formed. The ability of the repair polymerase pol β to incorporate dCTP at this position adds further support to this alternative, as misincorporation by this polymerase is undetectable even when the template is unfolded (Fig. 6E). Importantly, of the three reported intramolecular G-quadruplex structures formed by human telomeric sequence in the presence of KCl, one is a two-quartet structure in which this guanine nucleotide (i.e., the 3′ most G in a 5′-GGGTTAGGGTTAGGGTTAGGG-3′ sequence) does not participate [28]. Although results here cannot definitively determine which of these mechanisms applies or predominates, we speculate that the observed preference for dCTP incorporation by some polymerases is caused by availability of the template guanine in a proportion of templates assuming this two quartet G-quadruplex structure, while misincorporation events catalyzed by translesion polymerases might occur in a (partially or wholly) templated or untemplated manner. Unlike situations where DNA lesions are present in the template, we cannot conclude that misincorporation is specifically coded for by the presence of an intramolecular G-quadruplex. However, blockage of replicative DNA synthesis when these structures are in the template could elicit recruitment of error-prone translesion polymerases and increase the likelihood of misincorporation. Importantly, when misincorporation occurs on an intramolecular G-quadruplex-forming substrate, further extension of the primer appears completely blocked by a stable G-quadruplex. However, subsequent unfolding of the G-quadruplex along with continued extension from the mismatched 3′ end of the daughter strand would potentially lead to mutagenesis. We modeled such a mutagenic process using a two-step protocol (Fig. 7B). Indeed, our results with human pol η indicated that adenine, thymine, or guanine misincorporated upon encountering an intramolecular G-quadruplex was subject to further extension upon destabilization of the G-quadruplex (Fig. 7C). At physiological temperatures, intramolecular G-quadruplexes likely exist in a dynamic equilibrium with unstructured single-stranded DNA [29], a concept also supported by the experiments presented here. Thus, our two-step protocol mimics misincorporation at an intramolecular G-quadruplex followed by a dynamic shift to an unstructured template. Taken together, our results suggest that potential recruitment of an error-prone translesion polymerase such as pol η or pol κ, to a site of replication blockage caused by an intramolecular G-quadruplex may result in nucleotide misincorporation. Subsequent template unfolding, occurring either spontaneously or enzymatically, would generate a mismatch that is subject to further extension by the same or a different translesion polymerase, thus promoting substitution mutations within G-quadruplex-forming sequences. Further investigation is needed to determine whether there is an elevated frequency of substitution mutations in G-quadruplex forming regions. Notably, we did not detect skipping over of intramolecular G-quadruplexes by any of the polymerases tested, as has been observed with translesion polymerases on templates with specific DNA lesions [35]. This is likely due to a more pronounced steric effect of intramolecular G-quadruplex structures that occupy and affect many more nucleotides than bulky DNA adducts.

Our observation that all polymerases tested were substantially inhibited if not altogether blocked by an intramolecular G-quadruplex suggests that DNA polymerases alone probably cannot appropriately deal with these structures during replication. Since the inability to properly deal with these structures might result in rampant genomic and telomeric instability, a mechanism probably exists within cells to resolve these potentially problematic structures. A number of helicases have been shown to disrupt G-quadruplexes *in vitro*, including the *S. cerevisiae* proteins Pif1 [26], [39], [42] and Sgs1 [44] and human proteins PIF1 [27], FANCJ [45], [46], BLM [47], and WRN [48], [49]. Importantly, the human diseases (Fanconi anemia, BS, and WS) resulting from deficiency of FANCJ, BLM, and WRN, respectively, all exhibit genomic instability that may be related, in part, to inability to resolve G-quadruplex structures [50] and deficiencies in Sgs1, WRN, and BLM have been linked to alterations in gene expression related to potential G quadruplex-forming sequences in transcriptional units [34], [51]. Although they do not possess helicase activity, RPA [52] and POT1 (with telomeric sequences) [53] have been shown to promote unfolding (or stabilize the unfolded state) of these structures; notably, some domains within RPA possess preferential binding toward G-quadruplexes [54]. Disruption of these structures during replication by one or more of these factors would permit unperturbed DNA synthesis and diminish their capacity to induce genetic change. In yeast, it appears that Pif1 function is necessary to suppress chromosome breakage and resulting genomic instability caused by G-quadruplex formation during replication of specific loci, with little or no role for Sgs1or Rrm3 [26], [37]. On the other hand, Opresko and colleagues have recently shown an increased deletion frequency within a region of a vector containing telomeric repeats in human cells silenced for WRN, an Sgs1 homolog [55]. These studies reinforce the idea that one or more helicases may resolve G-quadruplexes formed during replication to help suppress related chromosome fragility, but do not address the possible recruitment and action of translesion polymerases to these replication-blocking structures.

In summary, intramolecular G-quadruplexes present a formidable obstacle for both replicative and translesion DNA polymerases. Stalling or blockage of replication by these structures could cause replication collapse, leading to double-strand breakage that would promote chromosomal and telomeric instability. Alternatively, misincorporation by translesion polymerases potentially recruited to sites of replication blocked by G-quadruplexes could result in elevated mutation rates within these sequences. Further investigation is needed to elucidate completely the effects of G-quadruplex structures on replication dynamics and genetic change.

## Supporting Information

Table S1 **Oligonucleotides Used in this Study.**
(PDF)

Table S2 **Description of DNA Polymerases Used.**
(PDF)

Table S3 Intramolecular G-Quadruplexes Dramatically Inhibit Synthesis by Various Polymerases on 4×GGG/*P34 Substrates.
(PDF)

Table S4 **Incorporation of Individual Nucleotides on Unfolded and G-quadruplex-forming Substrates.**
(PDF)

Table S5 **Incorporation of Multiple Cytosines on Unfolded and G-quadruplex-forming Substrates.**
(PDF)

Figure S1 **Gel Migration of Intermolecular G-quadruplex Species versus Intramolecular G-quadruplex-containing and Unfolded Partial Duplex Substrates.** To generate intermolecular G-quadruplexes, 3×GGG/*P31 (0.3 nM) was incubated with 5.9 µM 3×GGG oligomer (lane 2) and 4×GGG/*P31 (0.3 nM) with 5.9 µM 4×GGG oligomer (lane 3), respectively, in 10 mM Tris (pH 8.0), 1 mM EDTA, and 1 M NaCl at 37°C for 42 h. 3×GGG/*P31 (lane 1) and 4×GGG/*P31 (lane 4) partial duplex substrates were prepared without additional oligonucleotides and at reduced NaCl concentration (75 mM). DNA products were separated by native PAGE (6%, 19∶1) at 4°C in 0.5× TBE with 75 mM NaCl in both the gel and running buffer.
(PDF)

Figure S2 **Effect of KCl Concentration on G-quadruplex Stability.** To examine G-quadruplex stability in KCl, Kexo^−^ (2.5–25 U/L) was incubated with 3×CCC/*P31 or 4×GGG/*P31 (0.2 nM) each in extension buffer with and 50 or 100 mM KCl at 37°C for 5 min. Positions of partial extension products indicating polymerase stalling are highlighted (between red lines) and the first four nucleotides of the template and relevant product sizes are indicated at right.
(PDF)

Figure S3 **Incorporation at 35 nt Position is Not Dependent Upon Polymerase Exonuclease Activity.** Exonuclease proficient or exonuclease deficient T4 polymerase was incubated with 4×GGG/*P31 or 3×GGG/*P31 (0.2 nM) in extension buffer with 75 mM KCl at 37°C for 5 min.
(PDF)

Figure S4 ***S. cerevisiae*** **Pol η is Blocked by an Intramolecular G-quadruplex.** In the presence of 75 mM KCl, a primer extension assay was carried out using 3×GGG/*P31 or ext-4×GGG/*P31 (0.2 nM) and *S. cerevisiae* Pol η (0.85–2.1 nM) at 37°C for 5 min. Brackets denote the position of partial extension products indicating polymerase stalling.
(PDF)

Figure S5 Human Pol η Misincorporates Adenine or Thymine Upon Encountering Intramolecular G-quadruplex on ext-4×GGG/*P31 Substrate. Primer extension was performed on 3×GGG/*P31 or ext-4×GGG/*P31 (0.4 nM each) using human pol η (0.51–2.1 nM) in extension buffer containing 75 mM KCl with dNTPs or dATP + dTTP (100 µM each) at 18°C for 5 min. The brackets highlight products generated by incorporation in relation to the initial guanine in the 3×GGG and 4×GGG templates. Markers (M) were generated as described in Figure 4.
(PDF)
