# Supplementary material for: Intramolecular Telomeric G-Quadruplexes Dramatically Inhibit DNA Synthesis by Replicative and Translesion Polymerases, Revealing their Potential to Lead to Genetic Change
Source: PLoS One. 2014 Jan 14;9(1):e80664. doi: 10.1371/journal.pone.0080664 (PMC3891601; doi:10.1371/journal.pone.0080664)
Supplement: Table S1 — Oligonucleotides Used in this Study. (PDF) [file pone.0080664.s001.pdf]

Supplemental Table 1. Oligonucleotides used in this study.

Primers

P31
5’-CACTGACTCCAGGAACTGGAGGATGCCTAGG-3’

P34
5’-CACTGACTCCAGGAACTGGAGGATGCCTAGGTAA-3’

Templates

3xCCC
5’-TAACCCTAACCCTAACCCTAACCTAGGCATCCTCCAGTTCCTGGAGTCAGTG-3’

3xGGG
5’-TTAGGGTTAGGGTTAGGGTTACCTAGGCATCCTCCAGTTCCTGGAGTCAGTG-3’

4xGGG
5’-AGGGTTAGGGTTAGGGTTAGGGTTACCTAGGCATCCTCCAGTTCCTGGAGTCAGTG-3’

ext-4xGGG
5’-CATTTCATTTTAGGGTTAGGGTTAGGGTTAGGGTTACCTAGGCATCCTCCAGTTCCTGGA-GTCAGTG-3’

Other

4xGGG[22]
5’-AGGGTTAGGGTTAGGGTTAGGG-3’
