# Supplementary material for: Intramolecular Telomeric G-Quadruplexes Dramatically Inhibit DNA Synthesis by Replicative and Translesion Polymerases, Revealing their Potential to Lead to Genetic Change
Source: PLoS One. 2014 Jan 14;9(1):e80664. doi: 10.1371/journal.pone.0080664 (PMC3891601; doi:10.1371/journal.pone.0080664)
Supplement: Table S2 — Description of DNA Polymerases Used. (PDF) [file pone.0080664.s002.pdf]

Supplemental Table 2. Description of DNA Polymerases Used.

**Processive**		Klenow exo$^-$	Fragment of *E. coli* DNA Pol I; DNA repair polymerase; lacks exo
		T4 DNA Polymerase	T4 phage replicative polymerase; possesses 3’ to 5’ exo activity
		Human Pol δ	Lagging strand replicative polymerase; 3’ to 5’ exo activity [a]
**Distributive**	**Translesion**	Human Pol η	Y-family translesion polymerase; responsible for bypass of CPDs [a]
		S. cerevisiae Pol η	Y-family translesion polymerase; responsible for bypass of CPDs [b]
		Human Pol κ	Acts at abasic sites; low fidelity [a]
		Human Pol μ	Located in peripheral lymphoid tissues; potential hypermutase [a]
	**Other**	Human Pol β	Gap filling polymerase; involved in base excision repair [a]

[a] Hubscher, U., Maga, G., and Spadari, S. (2002) Eukaryotic DNA Polymerases. *Annu. Rev. Biochem.* **71**, 133-163

[b] Johnson, R.E., Prakash, S., and Prakash, L. (1999) Efficient bypass of a thymine-thymine dimer by yeast DNA polymerase, Pol eta. *Science* **283**, 1001-1004
