# Supplementary material for: Intramolecular Telomeric G-Quadruplexes Dramatically Inhibit DNA Synthesis by Replicative and Translesion Polymerases, Revealing their Potential to Lead to Genetic Change
Source: PLoS One. 2014 Jan 14;9(1):e80664. doi: 10.1371/journal.pone.0080664 (PMC3891601; doi:10.1371/journal.pone.0080664)
Supplement: Table S3 — Intramolecular G-Quadruplexes Dramatically Inhibit Synthesis by Various Polymerases on 4×GGG/*P34 Substrates. (PDF) [file pone.0080664.s003.pdf]

Supplemental Table 3. Intramolecular G-Quadruplexes Dramatically Inhibit Synthesis by Various Polymerases on ext-4xGGG/*P34 Substrates.

		3xGGG	4xGGG
**Pol η**	**34-35 nt**	15.5[a]	60.7
	**>35 nt**	84.5	39.3
**Pol κ**	**34-35 nt**	18.5	54.9
	**>35 nt**	81.5	45.1
**Pol μ**	**34-35 nt**	27.4	56.3
	**>35 nt**	72.6	43.7
**Pol β**	**34-35 nt**	16.7	42.5
	**>35 nt**	83.3	57.5

[a]All values, representing the percentage of total products in each reaction, were derived from Figure 6.
