# Supplementary material for: Intramolecular Telomeric G-Quadruplexes Dramatically Inhibit DNA Synthesis by Replicative and Translesion Polymerases, Revealing their Potential to Lead to Genetic Change
Source: PLoS One. 2014 Jan 14;9(1):e80664. doi: 10.1371/journal.pone.0080664 (PMC3891601; doi:10.1371/journal.pone.0080664)
Supplement: Table S4 — Incorporation of Individual Nucleotides on Unfolded and G-quadruplex-forming Substrates. (PDF) [file pone.0080664.s004.pdf]

Supplemental Table 4. Incorporation[a] of Individual Nucleotides on Unfolded and G-quadruplex-forming Substrates.

		3xGGG (unfolded)				4xGGG (G-quadruplex)			
		**dATP**	**dTTP**	**dGTP**	**dCTP[b]**	**dATP**	**dTTP**	**dGTP**	**dCTP[b]**
**Pol η**	**34 nt**	71.8	57.4	70.0	14.3	91.0	92.9	94.5	44.9
	**35 nt**	28.2	42.6	30.0	3.4	9.0	7.1	5.5	25.7
**Pol κ**	**34 nt**	82.6	41.3	71.4	17.3	97.3	99.2	95.6	65.5
	**35 nt**	17.4	58.7	28.6	6.1	6.6	4.4	8.5	14.0
**Pol μ**	**34 nt**	99.9	98.2	98.6	19.7	92.3	96.4	99.6	40.5
	**35 nt**	N.D.	N.D.	N.D.	5.0	7.7	3.6	N.D.	16.9
**Pol β**	**34 nt**	99.9	100	99.3	9.7	99.0	98.0	--------	21.2
	**35 nt**	N.D.	N.D.	N.D.	3.0	N.D.	N.D.	--------	21.1

[a]Values, expressed as percentages of the total products present in individual reactions, were derived from Figure 6. N.D. = below minimum detection limit.
[b]Substantial production of longer (36-39 nt) products was observed using dCTP (see Supplemental Table 5).
