# Supplementary material for: Intramolecular Telomeric G-Quadruplexes Dramatically Inhibit DNA Synthesis by Replicative and Translesion Polymerases, Revealing their Potential to Lead to Genetic Change
Source: PLoS One. 2014 Jan 14;9(1):e80664. doi: 10.1371/journal.pone.0080664 (PMC3891601; doi:10.1371/journal.pone.0080664)
Supplement: Table S5 — Incorporation of Multiple Cytosines on Unfolded and G-quadruplex-forming Substrates. (PDF) [file pone.0080664.s005.pdf]

**Supplementary Table 5. Incorporation of Multiple Cytosines on Unfolded and G-quadruplex-forming Substrates.**

| | | 3xGGG | 4xGGG |
|---|---|---|---|
| Pol η | 34 nt | 14.3[a] | 44.9 |
| | 35 nt | 3.4 | 25.7 |
| | 36 nt | 4.2 | 6.3 |
| | 37 nt | 44.9 | 15.6 |
| | 38 nt | 26.5 | 6.2 |
| | 39 nt | 6.9 | 1.2 |
| Pol κ | 34 nt | 17.3 | 65.5 |
| | 35 nt | 6.1 | 14.0 |
| | 36 nt | 5.6 | 3.6 |
| | 37 nt | 48.5 | 10.7 |
| | 38 nt | 14.8 | 4.0 |
| | 39 nt | 7.7 | 2.2 |
| Pol μ | 34 nt | 19.7 | 40.5 |
| | 35 nt | 5.0 | 16.9 |
| | 36 nt | 12.4 | 13.8 |
| | 37 nt | 55.0 | 26.0 |
| | 38 nt | 7.9 | 2.9 |
| | 39 nt | N.D. | N.D. |
| Pol β | 34 nt | 9.7 | 21.2 |
| | 35 nt | N.D. | 21.1 |
| | 36 nt | 4.5 | 7.9 |
| | 37 nt | 72.7 | 45.5 |
| | 38 nt | 10.2 | 3.9 |
| | 39 nt | N.D. | N.D. |

[a]Values, representing the percentage of total products for an individual reaction, were derived from Figure 6. N.D. = below the minimum detection limit.
