# Supplementary figures and images for: Intramolecular Telomeric G-Quadruplexes Dramatically Inhibit DNA Synthesis by Replicative and Translesion Polymerases, Revealing their Potential to Lead to Genetic Change
Source: PLoS One. 2014 Jan 14;9(1):e80664. doi: 10.1371/journal.pone.0080664 (PMC3891601; doi:10.1371/journal.pone.0080664)

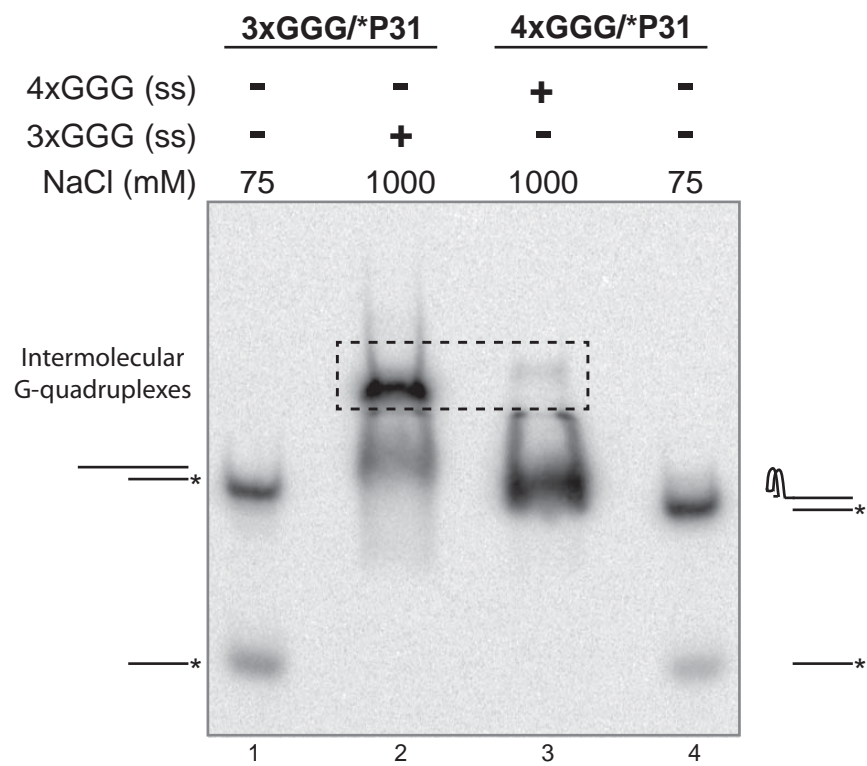

**Supplemental Figure 1**

Supplement: Figure S1 — Gel Migration of Intermolecular G-quadruplex Species versus Intramolecular G-quadruplex-containing and Unfolded Partial Duplex Substrates. To generate intermolecular G-quadruplexes, 3×GGG/*P31 (0.3 nM) was incubated with 5.9 µM 3×GGG oligomer (lane 2) and 4×GGG/*P31 (0.3 nM) with 5.9 µM 4×GGG oligomer (lane 3), respectively, in 10 mM Tris (pH 8.0), 1 mM EDTA, and 1 M NaCl at 37°C for 42 h. 3×GGG/*P31 (lane 1) and 4×GGG/*P31 (lane 4) partial duplex substrates were prepared without additional oligonucleotides and at reduced NaCl concentration (75 mM). DNA products were separated by native PAGE (6%, 19∶1) at 4°C in 0.5× TBE with 75 mM NaCl in both the gel and running buffer. (PDF) [file pone.0080664.s006.pdf]

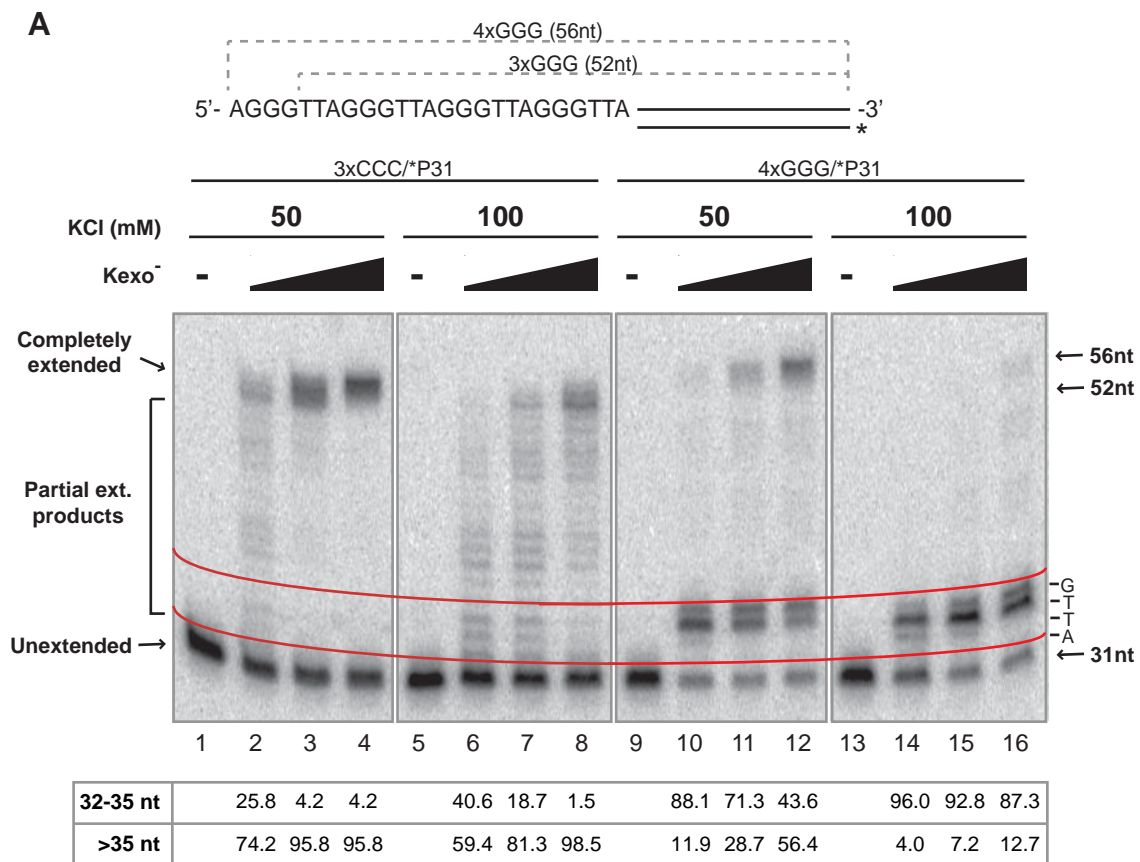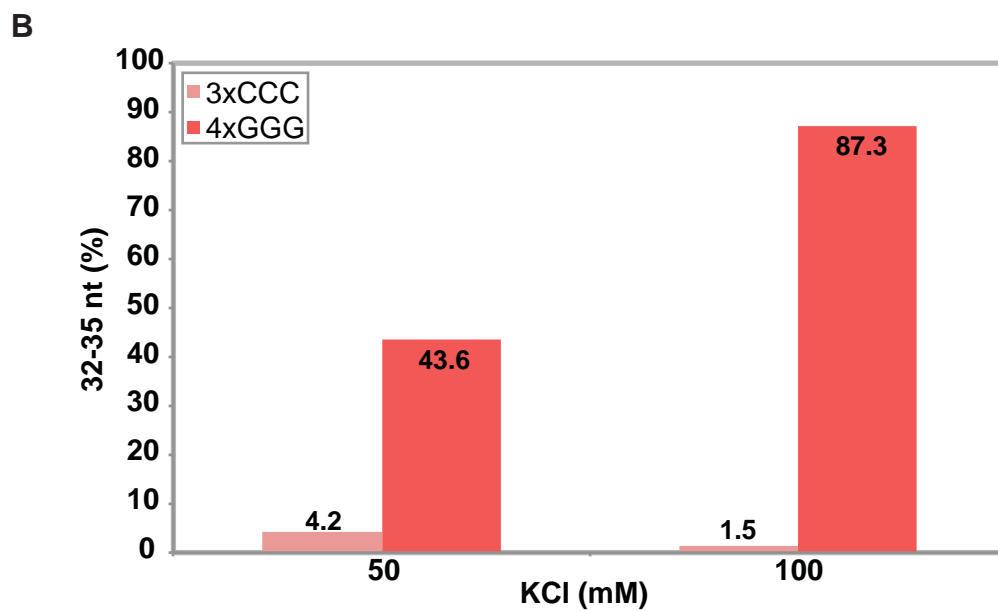

Supplemental Figure 1

Supplement: Figure S2 — Effect of KCl Concentration on G-quadruplex Stability. To examine G-quadruplex stability in KCl, Kexo− (2.5–25 U/L) was incubated with 3×CCC/*P31 or 4×GGG/*P31 (0.2 nM) each in extension buffer with and 50 or 100 mM KCl at 37°C for 5 min. Positions of partial extension products indicating polymerase stalling are highlighted (between red lines) and the first four nucleotides of the template and relevant product sizes are indicated at right. (PDF) [file pone.0080664.s007.pdf]

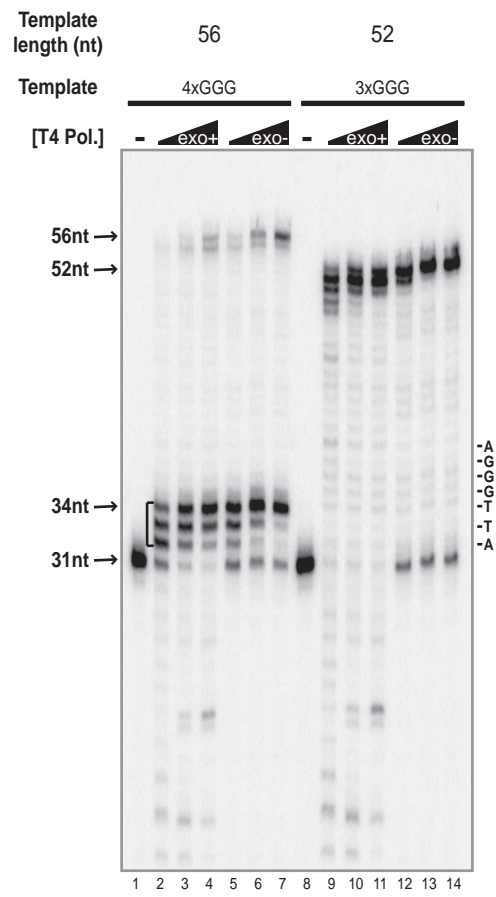

**Supplemental Figure 2**

Supplement: Figure S3 — Incorporation at 35 nt Position is Not Dependent Upon Polymerase Exonuclease Activity. Exonuclease proficient or exonuclease deficient T4 polymerase was incubated with 4×GGG/*P31 or 3×GGG/*P31 (0.2 nM) in extension buffer with 75 mM KCl at 37°C for 5 min. (PDF) [file pone.0080664.s008.pdf]

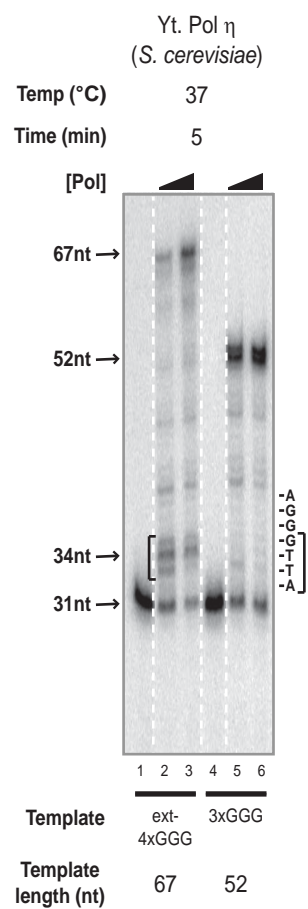

**Supplemental Figure 3**

Supplement: Figure S4 — S. cerevisiae Pol η is Blocked by an Intramolecular G-quadruplex. In the presence of 75 mM KCl, a primer extension assay was carried out using 3×GGG/*P31 or ext-4×GGG/*P31 (0.2 nM) and S. cerevisiae Pol η (0.85–2.1 nM) at 37°C for 5 min. Brackets denote the position of partial extension products indicating polymerase stalling. (PDF) [file pone.0080664.s009.pdf]

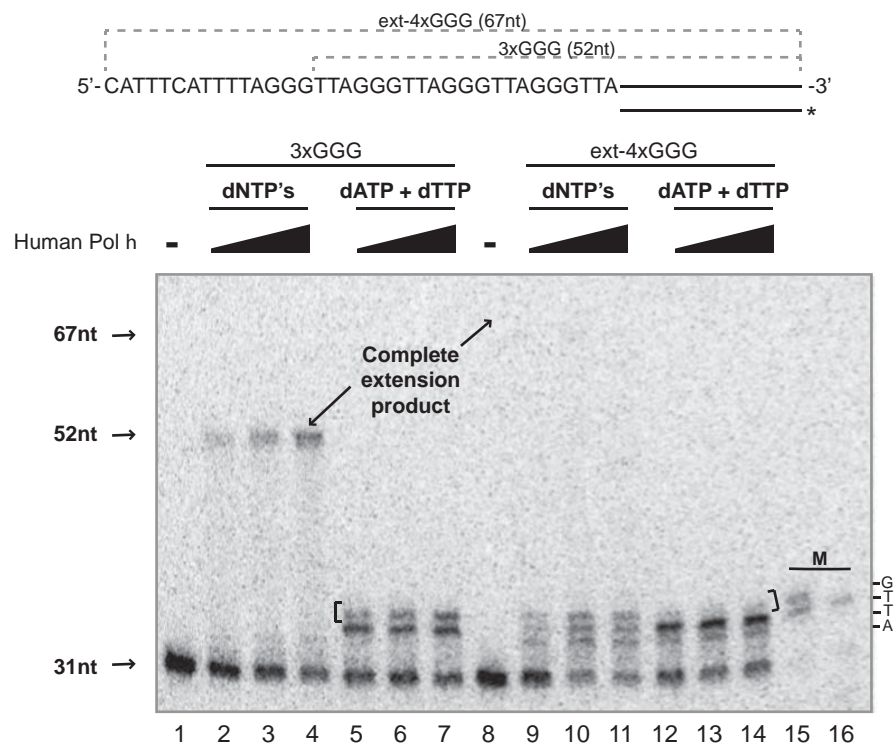

**Supplemental Figure 4**

Supplement: Figure S5 — Human Pol η Misincorporates Adenine or Thymine Upon Encountering Intramolecular G-quadruplex on ext-4×GGG/*P31 Substrate. Primer extension was performed on 3×GGG/*P31 or ext-4×GGG/*P31 (0.4 nM each) using human pol η (0.51–2.1 nM) in extension buffer containing 75 mM KCl with dNTPs or dATP + dTTP (100 µM each) at 18°C for 5 min. The brackets highlight products generated by incorporation in relation to the initial guanine in the 3×GGG and 4×GGG templates. Markers (M) were generated as described in Figure 4. (PDF) [file pone.0080664.s010.pdf]
